# Nucleophilic cleavage of C–F bonds by Brønsted base for rapid synthesis of fluorophosphate materials

**DOI:** 10.1093/nsr/nwaf020

**Published:** 2025-01-21

**Authors:** Qingfeng Fu, Zihao Chang, Peng Gao, Wang Zhou, Hongliang Dong, Peifeng Huang, Aiping Hu, Changling Fan, Peitao Xiao, Yufang Chen, Jilei Liu

**Affiliations:** College of Materials Science and Engineering, Hunan Joint International Laboratory of Advanced Materials and Technology for Clean Energy, Hunan Province Key Laboratory for Advanced Carbon Materials and Applied Technology, Hunan University, Changsha 410082, China; State Key Laboratory of Advanced Design and Manufacturing for Vehicle Body, Hunan University, Changsha 410082, China; College of Materials Science and Engineering, Hunan Joint International Laboratory of Advanced Materials and Technology for Clean Energy, Hunan Province Key Laboratory for Advanced Carbon Materials and Applied Technology, Hunan University, Changsha 410082, China; College of Materials Science and Engineering, Hunan Joint International Laboratory of Advanced Materials and Technology for Clean Energy, Hunan Province Key Laboratory for Advanced Carbon Materials and Applied Technology, Hunan University, Changsha 410082, China; Center for High Pressure Science and Technology Advanced Research, Shanghai 201203, China; State Key Laboratory of High Performance Ceramics and Superfine Microstructure, Shanghai Institute of Ceramics, Chinese Academy of Sciences, Shanghai 201899, China; State Key Laboratory of Advanced Design and Manufacturing for Vehicle Body, Hunan University, Changsha 410082, China; College of Materials Science and Engineering, Hunan Joint International Laboratory of Advanced Materials and Technology for Clean Energy, Hunan Province Key Laboratory for Advanced Carbon Materials and Applied Technology, Hunan University, Changsha 410082, China; College of Materials Science and Engineering, Hunan Joint International Laboratory of Advanced Materials and Technology for Clean Energy, Hunan Province Key Laboratory for Advanced Carbon Materials and Applied Technology, Hunan University, Changsha 410082, China; College of Aerospace Science and Engineering, National University of Defense Technology, Changsha 410073, China; College of Aerospace Science and Engineering, National University of Defense Technology, Changsha 410073, China; College of Materials Science and Engineering, Hunan Joint International Laboratory of Advanced Materials and Technology for Clean Energy, Hunan Province Key Laboratory for Advanced Carbon Materials and Applied Technology, Hunan University, Changsha 410082, China

**Keywords:** fluorophosphate compounds, C–F bond, polytetrafluoroethylene, potassium-ion battery

## Abstract

Fluorochemicals are a rapidly expanding class of materials used in a variety of fields including pharmaceuticals, metallurgy, agrochemicals, refrigerants, and in particular, alkali metal ion batteries. However, achieving one-step synthesis of pure fluorophosphate compounds in a well-controlled manner remains a formidable challenge due to the volatilization of fluorine during the heat treatment process. One feasible method is to cleave the C–F bond in polytetrafluoroethylene (PTFE) during synthesis to create a fluorine-rich atmosphere and strongly reducing environment. However, the inert nature of the C–F bond in PTFE presents a significant obstacle, as it is the strongest single bond in organic compounds. To address this predicament, we propose a fluorine-compensating strategy that involves cleavage of the C–F bonds by nucleophilic S_N_2-type reactions of Brønsted base (ammonia) enabling fluorine compensation. The decomposed products (NH_2_· and C·) also result in the formation of micropores (via NH_3_ escape) and *in-situ* carbon coating (via C· polymerization). The resultant cathode delivers a superior potassium storage capability including high rate performance and capacity retention. This contribution not only overcomes the obstacles associated with the inert C–F bond in fluororesin, but also represents a significant step forward in the development of fluorine-containing compounds.

## Teaser

A novel fluorine-compensating strategy that involves cleavage of the CF bonds by nucleophilic SN2-type reactions of Brønsted base (ammonia) is proposed for synthesizing high-purity fluorophosphate materials compared to previous highly toxic HF fluorolytic routes, which enabling the rapid and large-scale production of the fluorophosphate compounds with commercial application prospects.

## INTRODUCTION

Fluorochemicals are widely used in various fields including the metallurgical industry, pharmaceuticals, fluoropolymers, agrochemicals, and batteries, due to their high thermal stability and inertness [1–6]. In the field of batteries, fluorophosphate cathode materials (such as KVPO_4_F) have attracted considerable attention due to their robust three-dimensional framework and intriguing induced effects [7–12]. These structural features facilitate K^+^ diffusion, offer more K^+^ storage sites and high working potential (∼4.1 V), resulting in higher energy and power densities than other material systems [7,12–18]. However, the synthesis of phase-pure fluorophosphate compounds remains a challenging task due to the high volatility of fluorine during heat treatment, which makes it difficult to precisely control the phase purity and fluorine-to-oxygen ratio [12,19,20]. Therefore, the development of efficient and scalable methods to synthesize phase-pure fluorophosphate is highly desirable.

Over the past two decades, significant progress has been made in the synthesis of phase-pure fluorophosphate materials using a variety of synthetic techniques [9,10,19,21,22]. Despite these advances, the rapid synthesis of high purity fluorophosphates remains a significant challenge. The main research challenge lies in the volatility of elemental fluorine during precursor solution preparation and subsequent calcination steps, which can result in fluorine-deficient phases and impurities in the material (Fig. S1) [19]. Therefore, the synthesis of fluorophosphate is predominantly carried out using highly toxic liquid-phase fluorolytic routes and complex carbon thermal reduction (CTR) [23–26]. Liquid-phase fluorolytic routes require the use of HF and NH_4_F, both of which are highly toxic. Their widespread use in industry poses an immediate threat to public safety, making these methods unsuitable for further industrialization. The carbothermal reduction reaction involves two consecutive heat-treatment steps (precursors → VPO_4_/C → A*_x_*V*_y_*(PO_4_)*_z_*F*_a_* (A = Li, Na, K), Fig. S2), which are complex, time-consuming, step-by-step, solid-phase synthesis procedures which together with high energy consumption pose obstacles to large-scale industrialization. Therefore, it is of utmost importance to achieve a scalable and one-step synthesis approach for producing phase-pure fluorophosphate materials.

**Figure 1.**
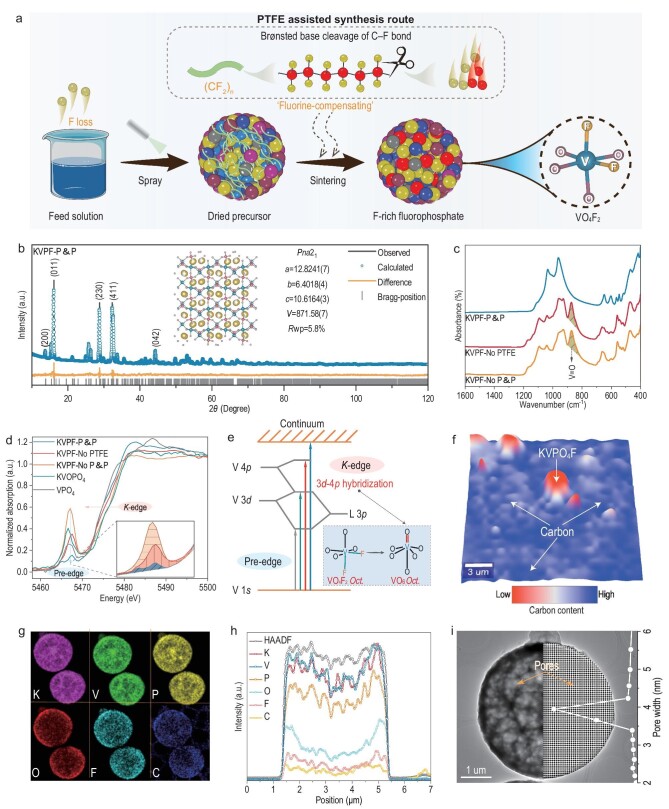
Structural analysis of the KVPO_4_F. (a) Schematic illustration of the PTFE assisted synthesis route with the fluorine-compensating strategy. (b) Rietveld refinement of KVPF-P&P. (c) FTIR of the KVPF-P&P, KVPF-No PTFE, and KVPF-No P&P. Vanadium *K*-edge (d) XANES spectra of KVPF-P&P, KVPF-No PTFE, and KVPF-No P&P. (e) Local environment of vanadium in VO_4_F_2_ and VO_6_. (f) Raman mapping of KVPF-P&P. (g) STEM-EDX mapping and (h) line scan of KVPF-P&P. (i) STEM of KVPF-P&P (inset: the pore size distribution of the KVPF-P&P sample).

Introducing an additional fluorine source (such as polytetrafluoroethylene (PTFE), (CF_2_)_n_) into the raw materials has emerged as an attractive strategy that can create a fluorine-rich atmosphere and provided *in-situ* carbon coating (also serving as reducing agent) in synthesis processes, offering a potential route for preparing phase-pure fluorophosphate. More importantly, the synergistic utilization of both fluorine and carbon components derived from PTFE not only enables effective degradation of PTFE waste but also establishes a closed-loop strategy for integrated resource recovery. Although this is a very attractive idea, cleaving the highly polar C–F bond in PTFE has proved difficult, largely because the C–F bond is the strongest single covalent bond in organic chemistry with a high bond energy of 485 kJ mol^–1^ (Figs S3 and S4) [27–30]. With these challenges in mind, we sought to develop a new fluorine-compensating strategy via cleaving the C–F bonds by nucleophilic attack of Brønsted base (ammonia), which enables efficient synthesis of fluorophosphate materials and also highlights the universality of the new synthesis method (Fig. 1a). The one-step synthesized phase-pure fluorophosphate (take KVPO_4_F as an example) exhibits high reversible capacity (94.2 mAh g^−1^ at 0.2C), superior rate capability (53.2 mAh g^−1^ at 20C) and good cyclability (retaining 55.4% of its initial capacity after 1000 cycles at 5C), which can be attributed to its porous microsphere structure, high electronic conductivity and surface coated carbon that leads to enhanced reaction kinetics, thus making it a promising cathode material for potassium-ion batteries (PIBs). This fluorine-compensating strategy, developed for the rapid synthesis of fluorophosphate materials, not only offers potential extensions to other fluorochemicals, but also heralds a novel route to their rapid, large-scale commercial production.

## RESULTS

### Synthesis and structure analysis

KVPO_4_F porous microspheres (KVPF-P&P) were synthesized via an optimized fluorine-compensating spray-dry process (for details, see Materials and Methods in Supplementary Material). To highlight the significant role of PTFE in the formation of pure phase KVPO_4_F, we also synthesized two types of KVPO_4_F without PTFE addition in feed solution, namely KVPF-No P&P obtained without any polyvinylpyrrolidone (PVP)/PTFE addition, and KVPF-No PTFE obtained by PVP addition alone (Fig. S5). Powder X-ray diffraction (PXRD) revealed the absence of any impurities in the KVPF-P&P, and crystal symmetry of the compound is well indexed to an orthorhombic space group (*Pna*2_1_) with *a* = 12.8241(7) Å, *b* = 6.4018(4) Å, *c* = 10.6164(3) Å, and *V* = 871.58(7) Å^3^ (Fig. 1b). All of the V^4+^ in the precursor is reduced to V^3+^ in the KVPF-P&P after sintering, as evidenced by the absence of V^4+^=O peaks at ∼886 cm^–1^ in the Fourier transform infrared (FTIR) spectra (Fig. 1c) [12]. The V^3+^ oxidation state in the KVPF-P&P is further validated by the V *K*-edge X-ray absorption near edge spectra (XANES) (Fig. 1d), which contains a weak pre-edge peak at 5460 and 5470 eV further supporting the existence of quasi-symmetric V^3+^O_4_F_2_ octahedral coordination and demonstrating a significant substitution of O with F [12,31,32]. The chemical composition of the KVPF-P&P was found to be 0.84K : 1.00V : 0.82P : 1.04F based on the inductively coupled plasma and ion selective electrode (ICP-ISE) test, indicating that the empirical formula for KVPF-P&P is close to KVPO_4_F. Scanning and transmission analytical electron microscopy (STEM-EDX) analysis clearly shows that the fluorine and carbon are uniformly distributed throughout the KVPF-P&P microspheres (Fig. 1g and h; Fig. S6). Carbon–sulfur analyzer data (Fig. S7g) and Raman mapping (Fig. 1f) confirms the existence of a high carbon content (4.44 wt%) and high homogeneity of the carbon signal observed in KVPF-P&P over large areas, indicating that the C–F bond cleavage is achieved via PTFE decomposition.

The important role of PTFE is further evaluated via analyzing KVPF-No P&P and KVPF-No PTFE. For samples prepared without PTFE, layered K_3_V_3_(PO_4_)_4_ impurity (ICDD card No. 83-1055) was identified along with the KVPO_4+_*_x_*F_1-_*_x_* phase (Fig. S8), and the composition is determined to be oxygen-rich KVPO_4+_*_x_*F_1-_*_x_* phase from FTIR analysis (Fig. 1c). The fingerprint of the V^4+^=O signal at ∼886 cm^–1^ is detected in the FTIR spectrum, corroborates the presence of V^4+^O_6_ octahedra, indicating insufficient fluorine for oxygen substitution [12]. Further supports are derived from XANES analysis, in which there is a strong pre-edge peak at 5465 eV that is a characteristic signature of the excitation from noncentrosymmetric V^4+^O_6_ octahedron environments due to V 3*d*-4*p* hybridization (Fig. 1e) [7,12,31]. The bulk K: V: P: F ratios for KVPF-No PTFE and KVPF-No P&P were determined by ICP-ISE measurements to be 0.83K: 1.00V: 0.96P: 0.23F and 0.77K: 1.00V: 0.93P: 0.26F, respectively. All these are attributed to fluorine loss upon the high temperature calcination process (HF, F_2_ gas), highlighting why fluorine compensation is highly desirable. Note that high-resolution transmission electron microscopy (HRTEM) (Figs S9 and S10) and carbon–sulfur analyzer data (Fig. S7) indicate that there is no detectable carbon in KVPF-No P&P (0.13 wt%), whereas the KVPF-No PTFE sample exhibits an irregular carbon coating (2.16 wt%). These differences indicate that the carbon was decomposed from polyvinylpyrrolidone (PVP) rather than oxalic acid, a departure from the traditional view.

The sphere-like morphology and porous structure of KVPF-P&P are revealed by the scanning electron microscope (SEM) and STEM images (Fig. S6a and b), as well as the nitrogen adsorption-desorption isotherms (Fig. S7), which is likely due to the decomposition of vanadyl oxalate and PTFE into gaseous substances during calcination and therefore is beneficial to the infiltration of electrolytes (Fig. S11). KVPF-No P&P shows a similar spherical morphology with a more compact structure (Fig. S12), and KVPF-No PTFE has sporopollenin-like morphology features which are due to PVP-induced volume shrinkage resulting from the effect of surfactant (Figs S13 and S14) [33]. Notably, the PVP in the feed solution serves as a surface dispersant to disperse the PTFE emulsion and prevent the PTFE particles from aggregating (Fig. S15). The microporous structural features are further confirmed by the presence of a hysteresis loop in KVPF-P&P and KVPF-No PTFE, but not in KVPF-No P&P (Fig. S7). The specific surface area of KVPF-P&P is calculated to be 24.0 m^2^ g^–1^, which is ∼3 times larger than KVPF-No PTFE (8.8 m^2^ g^–1^) and ∼2 times larger than KVPF-No P&P (13.2 m^2^ g^–1^). Figure S16 show particle size distributions of three samples determined by a laser particle size analyzer, with average particle sizes (D50) of 4.25 μm for KVPF-P&P, 7.56 µm for KVPT-No PTFE, and 6.40 µm for KVPF-No P&P, which are consistent with the results obtained from BET and SEM analysis, indicating facile electrolyte penetration and shortened K^+^ diffusion pathways in the KVPF-P&P electrode [34].

### Key role of Brønsted base for cleavage of C–F bonds in PTFE

**Figure 2.**
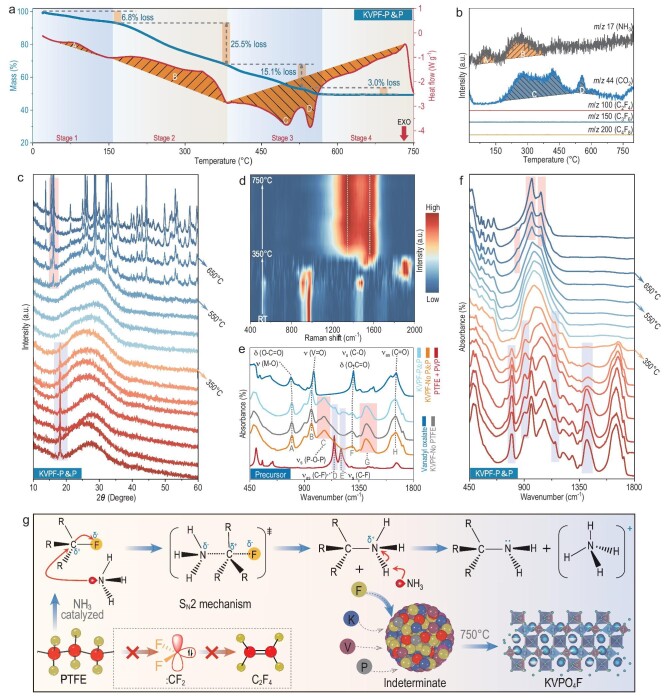
Key role of the Brønsted base for cleavage of C–F bonds in PTFE. (a) TG/DSC curves and (b) corresponding mass spectra of the KVPF-P&P precursor. (c) Temperature-dependent PXRD patterns and (d) temperature-dependent Raman spectra of the KVPF-P&P precursor at different calcination temperatures. (e) FTIR spectra of the KVPF-P&P, KVPF-No PTFE, KVPF-No P&P precursors, PTFE + PVP, and vanadyl oxalate. (f) Temperature-dependent FTIR spectra of the KVPF-P&P precursor at different calcination temperatures. (g) Mechanism for the synthesis of KVPF-P&P.

To explore the synthetic reaction mechanism, systematically temperature-dependent characterizations (thermogravimetry/mass spectrometry (TG/MS), PXRD, Raman, FTIR, *etc.*) are conducted. Optical images revealed a color change of KVPF-P&P precursors from green (RT to 100°C) to yellow (100°C to 300°C) and finally to black (>350°C), indicating a carbonization temperature of ∼350°C for KVPF-P&P precursors (Fig. S17), which is also verified by the TG-DSC curve. As illustrated in Fig. 2a, four distinct weight losses were observed in different temperature ranges: 30–150°C (∼6.8%, endothermic peak A) corresponding to water evaporation, 150–350°C (∼25.5%, endothermic peak B) attributed to vanadyl oxalate decomposition, 350–530°C (∼15.1%, exothermic peak C) associated with PVP/PTFE decomposition (carbonization), and 530–750°C (∼3.0%, exothermic peak D) signifying the formation of the final KVPO_4_F product. Temperature-dependent PXRD and Raman further confirmed the disappearance of the characteristic diffraction peaks of PTFE at 350°C, followed by a noncrystallisation carbonization process between 400°C and 500°C (Fig. 2c and d). At 500°C, a sharp (011) diffraction peak of KVPO_4_F (at ∼16°C) appeared, indicating the KVPO_4_F particles formed during the fluorination and carbonization process associated with the V-reduction and fluorine compensation at this stage. Figure 2e shows the FTIR spectra of vanadyl oxalate, KVPF-P&P, KVPF-No PTFE, KVPF-No P&P, and P&P precursors. The δ (O–C=O) (∼790 cm^–1^), υ_s_ (V–O) (∼958 cm^–1^), and υ_as_ (C=O) (∼1879 cm^–1^) of vanadyl oxalate, the υ_s_ (P–O–P) (∼1062 cm^–1^) and υ_s_ (N–H) (∼1410 cm^–1^) of NH_4_H_2_PO_4_ in KVPF-P&P precursors, and the υ_as_ (C–F) (∼1147 cm^–1^) and υ_s_ (C–F) (∼1201 cm^–1^) of PTFE were used to track the changes in chemical structure evolution during the synthesis process. From RT to 350°C (Fig. 2f), the δ (O–C=O), υ_s_ (V–O), and υ_as_ (C=O) in vanadyl oxalate disappeared, indicating the decomposition of vanadyl oxalate to form VO_2_/CO_2_. Simultaneously, decomposed NH_4_H_2_PO_4_ in the precursor reacts with potassium to form ammonia (NH_3_) gas and K–P–O inorganic base complexes, as evidenced by the TG-MS and FTIR (Fig. 2b and f) [35–37]. As the nucleophile NH_3_ approaches the electrophilic carbon from the back side (S_N_2 reaction mechanism), a new C–NH_3_ bond is formed and old C–F bond breakage occur simultaneously, resulting in the R_3_C–NH_3_ product [38–41]. Then an ammonia molecule removes a hydrogen ion from the –NH_3_^+^ group, forming an ammonium ion and an amine. Subsequently, homolysis of the amine at high temperatures produces free C· and ·NH_2_ radicals, leading to the formation of carbon, NH_3_ and N_2_ (Fig. S18). In addition, DFT calculations showed that the thermodynamic Gibbs free energy (ΔG) was negative, further demonstrating the spontaneous nature of the PTFE with NH_3_ reaction (Fig. S19). The schematic diagram of KVPF-P&P synthetic procedures is demonstrated in Fig. 2g. In order to verify this reaction mechanism, a validation experiment was also carried out (Fig. S20) and it was shown that PTFE can undergo C–F bond cleavage under an ammonia atmosphere, which leads to the formation of carbon materials. This S_N_2 reaction avoids the decomposition of PTFE into CF_2_ and finally the formation of C_2_F_4_ (no signal was detected in TG-MS, Fig. 2b), realizing the full utilization of F and C atoms in the synthesis process.

A different structural evolution is observed in both KVPF-No PTFE and KVPF-P&P samples. The different color changes (gradually from green to yellow-brown) of KVPF-No P&P precursors show that no obvious carbonization occurred during the calcination process (Fig. S21), indicating that the citric acid and vanadyl oxalate cannot be directly used as a carbon source (Fig. S22). Despite the color changes of KVPF-No PTFE being similar to the process of KVPF-P&P (Fig. S23), a much higher carbonization temperature (∼400°C, comes from the decomposition of PVP) is identified for KVPF-No PTFE with respect to that of KVPF-P&P, which is consistent with the TG curve of PVP (Fig. S22). And oxygen-rich KVPO_4+_*_x_*F_1-_*_x_* appear at as high as 700°C accompanied by numerous impurities in KVPF-No P&P and KVPF-No PTFE samples, as evidenced from PXRD and FTIR results (Figs S24 and S25). Furthermore, FTIR showed a strong V^4+^=O peak at 886 cm^–1^, which reflects the fact KVPF-No P&P and KVPF-No PTFE undergoes insufficient carbonization and fluorination in the synthesis process. These results lend further support to the key role of PTFE in the catalysed synthesis of pure phase fluorophosphate materials: (i) Completely breaking the C–F bond can create a fluorine-rich atmosphere, enabling fluorine compensation and subsequently regulating the fluorine-oxygen ratio within the material; (ii) PTFE can also serve as a reducing agent, facilitating the reduction of the valence state V from +4 to +3 and allowing *in-situ* carbon coating of KVPO_4_F during the synthesis process; (iii) Homolysis of the amine (the S_N_2 reaction product) decomposes into NH_3_ and N_2_ gas during calcination, resulting in the formation of a porous structure that facilitates electrolyte infiltration. To further confirm this, we designed an intuitive cross-validation experiment (detailed in Materials and Methods in the Supplementary Material and Fig. S26). After introducing the commercial PTFE powder into the KVPF-No P&P precursor, the resulting KVPF-No P&P shows diffraction peaks that were all coincident with the pure KTiOPO_4_-type phase, again demonstrating the important role of PTFE in the synthesis of high phase pure fluorophosphate materials.

In addition, we have successfully prepared other fluorophosphate compounds (LiVPO_4_F and Na_3_V_3_(PO_4_)_2_F_3_) via this fluorine-compensating strategy. Detailed characterization by PXRD and SEM confirmed the formation of the triclinic structure of LiVPO_4_F (Fig. S27a, ICDD card No. 42-1412) and the tetragonal structure of Na_3_V_3_(PO_4_)_2_F_3_ (Fig. S27b, ICDD card No. 89-8485) without the presence of any impurity peaks, thus demonstrating the successful preparation of pure phases. This result again proves the universality of the fluorine-compensating strategy for the synthesis of pure fluorophosphate compounds.

### K^+^ storage performance

**Figure 3.**
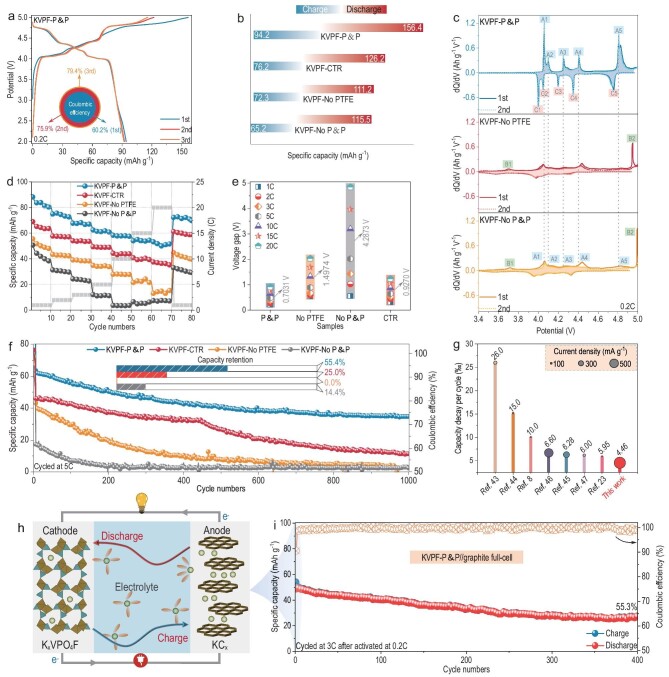
Electrochemical K^+^ storage performance. (a) Galvanostatic charging/discharging profiles of KVPF-P&P at 0.2C. (b) Illustration comparison for practical capacities of KVPF-P&P, KVPF-CTR, KVPF-No PTFE and KVPF-No P&P. (c) dQ/dV plots for the first two cycles at 0.2C. (d, e) Rate capability and corresponding voltage polarization. (f) Long-term cyclability at 5C. (g) Comparison of cycle performance of various cathode materials for PIBs. (h) Schematic diagram of KVPF-P&P//graphite full-cell. (i) Long-term cycle performance of KVPF-P&P//graphite full-cell at 3C.

Electrochemical investigations in both half-cell and full-cell configurations have confirmed the favorable effect of fluorine compensation on the performance of KVPO_4_F. Typical first three charge–discharge cycle curves at 0.2C show that the KVPF-P&P delivers an initial discharge capacity of 94.2 mAh g^–1^, which is significantly higher than those of KVPF-No PTFE (72.3 mAh g^–1^), KVPF-No P&P (65.2 mAh g^–1^) and KVPF-CTR (76.2 mAh g^–1^), demonstrating that the active materials are electrically well connected and efficiently participate in electrochemical depotassiation and potassiation (Fig. 3a and b; Fig. S28). Additionally, the corresponding differential capacity analysis (dQ/dV) curves clearly demonstrated that the KVPF-P&P electrodes exhibited five distinct oxidation/reduction peaks at 4.06/3.97 (A1/C1), 4.12/4.03 (A2/C2), 4.26/4.18 (A3/C3), 4.42/4.36 (A4/C4), and 4.82/4.72 V (A5/C5), corresponding to the highly reversible transformation between ${\mathrm{V}}_{cis}^{3 + }$↔${\mathrm{V}}_{cis}^{4 + }$ and ${\mathrm{V}}_{\textit{trans}}^{3 + }$↔${\mathrm{V}}_{\textit{trans}}^{4 + }$ (Fig. 3c and Fig. S29) [42]. In contrast, the KVPF-No P&P and KVPF-No PTFE electrodes exhibited broad and irreversible peaks (B1 and B2) in the same voltage range, indicating inferior electrochemical activity and unfavorable phase transitions caused by the presence of layered K_3_V_3_(PO_4_)_4_ impurity phase and its low electronic conductivity. Rate performance tests also reveal a significant capacity decay for KVPF-No P&P and KVPF-No PTFE, with only 14.0 and 7.4 mAh g^–1^ being maintained at a current rate of 20C (Fig. 3d), respectively. For KVPF-P&P, impressive high capacities of 88.2, 74.9, 67.7, 62.4, 58.3, 54.0 and 53.2 mAh g^–1^ were obtained at current rates of 1, 2, 3, 5, 10, 15 and 20C, respectively, highlighting the crucial role of its structural design advantage on high rate performance. The enhanced electrochemical reaction kinetics in KVPF-P&P was also magnified in the selected voltage polarization between charge and discharge plateaus at different rates as shown in Fig. 3e and Fig. S30. Clearly, KVPF-P&P shows a smaller voltage gap and polarization from 0.2 to 20C, indicating fast electrochemical reaction kinetics. Subsequent cycling tests at 1C further validate the superior reversibility of KVPF-P&P electrodes (Fig. S31), whereas KVPF-No P&P and KVPF-No PTFE electrodes exhibit consistent capacity decay and large voltage gap, indicating inferior cycling performance. Figures 3f and S32 further illustrate this disparity with an extended 5C cycle. The accelerated capacity degradation observed in the KVPF-No P&P and KVPF-No PTFE electrodes during cycling can be attributed to continuous structural collapse (Fig. S33). Remarkably, the KVPF-P&P electrode provides significantly improved stability, retaining 55.4% of the initial capacity for >1000 cycles. *Ex-situ* EIS and SEM observations confirm that the good cyclic stability of KVPF-P&P is due to superior structural stability over long-term cycling (Figs S33 and S34). The good cycling stability of KVPF-P&P significantly outperformed previous reports and other vanadium-containing polyanionic compounds (Fig. 3g and Table S1) [8,23,43–47]. To further investigate the practical applications, a prototype full cell was assembled by pairing the KVPF-P&P cathode with a pre-potassiated graphite anode (Fig. 3h). The full cell delivered a high discharge capacity of 87.3 mAh g^–1^ at 0.2C (based on the anode mass) and retained 55.3% of the capacity after 400 cycles at 3C (Fig. 3i and Fig. S35), demonstrating great practical feasibility.

### Improved K^+^ kinetics mechanism

**Figure 4.**
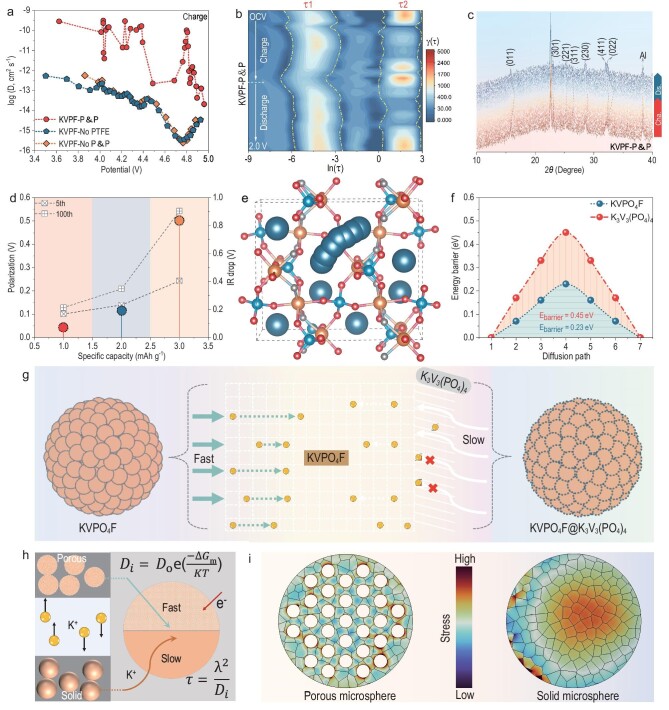
Improved K^+^ kinetics mechanism. (a) GITT determined K^+^ diffusion coefficients. (b) DRTs of KVPF-P&P. (c) *In-situ* PXRD pattern of KVPF-P&P. (d) The IR drop and polarization. (e) Schematic illustration of K^+^ migration path in KVPO_4_F. (f) K^+^ diffusion energy barriers calculated in KVPO_4_F and K_3_V_3_(PO_4_)_4_ structures and (g) corresponding K^+^ storage mechanism. (h) Schematic illustrations of the advantages of a porous microsphere. (i) Finite element simulation of the equivalent stress distribution in the cathode particles.

In order to highlight the advantages of the phase pure and structural features on electrochemical performance, a systematic kinetic analysis was carried out. KVPF-P&P retains the sharp and highly reversible redox peak shapes in cyclic voltammetry (CV) analysis at a scan rate ranging from 0.1 to 1.0 mV s^–1^ (Fig. S36), unambiguously confirming the ultra-fast charge-storage kinetics. Conversely, the redox peak shapes of KVPF-No P&P and KVPF-No PTFE diminish as the scan rate increases, indicating inadequate electron/ion transport kinetics within the electrodes due to the presence of a layered K_3_V_3_(PO_4_)_4_ impurity phase. In addition, the presence of some irreversible cathodic and anodic peaks (3.71/3.55 V) in KVPF-No P&P and KVPF-No PTFE indicates the presence of complicated irreversible phase transitions that lead to unsatisfactory rate performance. The fast K^+^ transport of KVPF-P&P was further confirmed by temperature-dependent electrochemical impedance spectra (EIS) measurements (Fig. S37). The calculated activation energy of KVPF-P&P was 10.9 kJ mol^–1^, much smaller than that of KVPF-No P&P (11.9 kJ mol^–1^) and KVPF-No PTFE (15.6 kJ mol^–1^). This observation is further supported by the K^+^ diffusion coefficient (*D_K_*) values obtained by GITT (Fig. 4a and Fig. S38). The average *D_K_* values in KVPF-P&P, namely 6.93 × 10^−11^ for the charging process and 1.39 × 10^−10^ cm^2^ s^–1^ for the discharging process, exceed those in KVPF-No P&P (7.68 × 10^−14^ for the charging process and 1.77 × 10^−13^ cm^2^ s^–1^ for the discharging process) and KVPF-No PTFE (7.52 × 10^−14^ for the charging process and 2.04 × 10^−13^ cm^2^ s^–1^ for the discharging process). Apart from *D_K_, in-situ* EIS analysis was also carried out to investigate the interfacial evolution during K^+^ extraction/insertion, as illustrated in Fig. S39. To distinguish the contributions of the charge transfer resistance (*R_ct_*) and solid interfacial layer (*R_SEI_*) processes within the high- to mid-frequency range, we employed distribution of relaxation time (DRT) deconvolution on each impedance spectrum [48–50]. The results are shown in Fig. 4b, where the integral areas of two distinct peaks ln(τ1) and ln(τ2) located at –6∼–3 and 0∼3 are assigned to the *R_SEI_* and *R_ct_*, respectively. The precise values for *R_SEI_* and *R_ct_* can be determined by fitting the DRT peaks. The evolution of the calculated *R_ct_* during the whole cycle is shown in Fig. S39g. Obviously, the *R_ct_* of the KVPF-P&P electrode is lower than that of the KVPF-No P&P and KVPF-No PTFE electrodes, indicating that the KVPF-P&P has lower energy barriers for ion and charge transfer.

Results for dQ/dV testing in 1C at different cycles can be used to reveal the capacity fading behaviour. Figure S40a shows that the KVPF-P&P electrode exhibits five identical peaks at voltages of 3.87, 3.93, 4.09, 4.25 and 4.66 V, demonstrating superior electrochemical activity. After 50 cycles, the intensities of almost all peaks are affected by the prolonged cycle and the peaks are either attenuated or merged. Nevertheless, the major peaks of KVPF-P&P are still present in the dQ/dV curve, confirming highly stable redox reactions and a highly reversible potassiation/depotassiation process. Besides, *in-situ* PXRD results reflect that the reversible potassiation/depotassiation of KVPF-P&P proceeds in only a single-phase reaction from KVPO_4_F to VPO_4_F (Fig. 4c), and also verified that KVPF-P&P demonstrated superior structural stability. In contrast, the KVPF-No P&P and KVPF-No PTFE electrodes display two low-intensity broad peaks at ∼3.86 and ∼4.09 V (Fig. S40b and c). As the number of cycles further increased, the intensity of the peak decreases and the peaks become broader or even disappear, indicating an inadequate redox reaction process. This phenomenon is even more pronounced in KVPF-No P&P due to the lack of carbon protection. Battery failure can be further understood by comparing the polarization of the voltage drop during the cycle (Fig. 4d). The KVPF-P&P electrode exhibits the smallest voltage hysteresis (0.169 V) at 1C, with a polarization of only 0.045 V after 50 cycles. In contrast, the KVPF-No P&P and KVPF-No PTFE electrodes demonstrate higher voltage polarizations (0.501 V for KVPF-No P&P and 0.115 V for KVPF-No PTFE) after 50 cycles, leading to a rapid decline in capacity.

Generally, the superior rate performance and cycling stability of KVPF-P&P in PIBs can be mainly attributed to the following aspects: (i) The phase-pure KVPF-P&P sample features an open and stable 3D framework structure, which facilitates ion diffusion along the *c*-axis channel with a low diffusion energy barrier, effectively enhancing the discharge capacity and rate performance. When a layered K_3_V_3_(PO_4_)_4_ impurity phase is present, it will undoubtedly disrupt the 3D K^+^ transport network in the KVPO_4_F structure, resulting in sluggish diffusion kinetics (Fig. 4g). DFT calculation results (Fig. 4e and f; Fig. S41) further suggest that the diffusion energy barrier of the polyanionic KVPO_4_F is 0.23 eV (with K^+^ migrating along the path K2→K1), which is much lower than that of the layered structure K_3_V_3_(PO_4_)_4_ (0.45 eV, with K^+^ migrating along the path K2→K1), contributing to significantly enhanced diffusion kinetics. (ii) The unique porous structure in KVPF-P&P not only accelerates ion diffusion and provides more effective sites for their adsorption and enhancing reaction kinetics, but also helps to alleviate volume expansion [20,51–54]. According to the Arrhenius relationship and Einstein's formula (Fig. 4h) [55–57], the porous structure can accelerate the solid-state diffusion of K^+^ and electrons due to the shortening of the diffusion distance and the reduced diffusion path for ion migration, undoubtedly significantly improving the rate capability and cycle stability. Furthermore, the porous structure could also offer enhanced mechanical stability by releasing stress concentration, particularly for large size K^+^ diffusion induced stresses that typically leads to significant volume changes during insertion and extraction. These merits are well supported from finite element method (FEM) simulation results (Fig. 4i and Fig. S42), in which the stress concentration in a KVPF-No P&P solid sphere increases progressively from the inside to the outside, culminating in the formation of a stress concentration point on the surface, showing a higher susceptibility to structural fracture, and resulting in poor cycle stability. Conversely, the maximum stress in the KVPF-P&P porous microsphere is lower than that in the KVPF-No P&P solid sphere, suggesting greater resistance to fracture and superior capacity retention. (iii) The interconnected amorphous carbon layers in KVPF-P&P accelerate electron transfer and reduce charge transfer resistance [53,58], further enhancing the rate capability. The electronic conductivity calculated by EIS shows that KVPF-P&P has a higher ionic conductivity (1.07 × 10^−2^ S cm^–1^) than that of KVPT-No PTFE (6.04 × 10^−8^ S cm^–1^) and KVPF-No P&P (3.44 × 10^−10^ S cm^–1^). According to percolation theory [59–62], a rapid increase in electrical conductivity occurs when the conductive carbon is wrapped on the surfaces of KVPO_4_F particles to form a 3D conductive network, which greatly reduce the contact internal resistance between particles, resulting in significantly reduced charge transfer resistance. All of these indicated that pure phase and 3D porous interconnected carbon networks could effectively improve electrochemical performance of KVPO_4_F, highlighting the importance of fluorine compensation.

## DISCUSSION

In summary, a simple, rapid and universal one-step spray-dry method for the synthesis of KVPO_4_F microspheres has been proposed via the introduction of an intriguing fluorine-compensating strategy. Systematic structural analysis revealed that the high nucleophilicity of ammonia leads to the cleavage of inert C–F bonds in PTFE, thus allowing for fluorine compensation in the synthesis process. Meanwhile, cleaving the C–F bonds in PTFE also generates large amounts of carbon and gas, leading to the formation of porous structures and *in-situ* carbon coatings. Benefiting from its pure phase, porous structures and *in-situ* carbon coatings, KVPO_4_F demonstrates superior rate capability and cyclability, attributed to the improved structural stability and diffusion kinetics. Importantly, several other fluorophosphate compounds have also been successfully synthesized using this fluorine-compensating strategy, highlighting the universality of this method. We hope that the findings of this study will pave the way for future improvements in the efficient synthesis of fluorophosphate in various fields of science and technology.

## Supplementary Material

nwaf020_Supplemental_File

## References

[1] Patel C, André-Joyaux E, Leitch JA et al. Fluorochemicals from fluorspar via a phosphate-enabled mechanochemical process that bypasses HF. Science 2023; 381: 302–6. 10.1126/science.adi1557 37471551

[2] Wang Y, Yang X, Meng YF et al. Fluorine chemistry in rechargeable batteries: challenges, progress, and perspectives. Chem Rev 2024; 124: 3494–589. 10.1021/acs.chemrev.3c00826 38478597

[3] Dolbier WR . Fluorine chemistry at the millennium. J Fluor Chem 2005; 126: 157–63. 10.1016/j.jfluchem.2004.09.033

[4] Isanbor C, O'Hagan D. Fluorine in medicinal chemistry: A review of anti-cancer agents. J Fluor Chem 2006; 127: 303–19. 10.1016/j.jfluchem.2006.01.011

[5] Zhang R, Shen P, Xiong Y et al. Bright, photostable and long-circulating NIR-II nanoparticles for whole-process monitoring and evaluation of renal transplantation. Natl Sci Rev 2024; 11: nwad286. 10.1093/nsr/nwad286 38213521 PMC10776353

[6] Tan S, Jiang Y, Ni S et al. Serrated lithium fluoride nanofibers-woven interlayer enables uniform lithium deposition for lithium-metal batteries. Natl Sci Rev 2022; 9: nwac183. 10.1093/nsr/nwac183 36381218 PMC9647010

[7] Fedotov SS, Khasanova NR, Samarin AS et al. AVPO_4_F (A = Li, K): a 4 V cathode material for high-power rechargeable batteries. Chem Mater 2016; 28: 411–5. 10.1021/acs.chemmater.5b04065

[8] Chihara K, Katogi A, Kubota K et al. KVPO_4_F and KVOPO_4_ toward 4 volt-class potassium-ion batteries. Chem Commun 2017; 53: 5208–11. 10.1039/C6CC10280H 28443861

[9] Recham N, Chotard JN, Dupont L et al. A 3.6 V lithium-based fluorosulphate insertion positive electrode for lithium-ion batteries. Nat Mater 2010; 9: 68–74. 10.1038/nmat2590 19946280

[10] Fedotov SS, Luchinin ND, Aksyonov DA et al. Titanium-based potassium-ion battery positive electrode with extraordinarily high redox potential. Nat Commun 2020; 11: 1484. 10.1038/s41467-020-15244-6 32198379 PMC7083823

[11] Nikitina VA, Fedotov SS, Vassiliev SY et al. Transport and kinetic aspects of alkali metal ions intercalation into AVPO_4_F framework. J Electrochem Soc 2017; 164: 6373–80. 10.1149/2.0531701jes

[12] Wernert R, Nguyen LHB, Petit E et al. Controlling the cathodic potential of KVPO_4_F through oxygen substitution. Chem Mater 2022; 34: 4523–35. 10.1021/acs.chemmater.2c00295

[13] Ahsan MT, Ali Z, Usman M et al. Unfolding the structural features of NASICON materials for sodium-ion full cells. Carbon Energy 2022; 4: 776–819. 10.1002/cey2.222

[14] Ahsan MT, Qiu DP, Ali Z et al. Unraveling the fast Na diffusion kinetics of NASICON at high voltage via high entropy for sodium-ion battery. Adv Energy Mater 2024; 14: 2302733. 10.1002/aenm.202302733

[15] Wang L, Zhang S, Li N et al. Prospects and challenges of practical nonaqueous potassium-ion batteries. Adv Funct Mater 2024; 34: 2408965. 10.1002/adfm.202408965

[16] Zhang Z, Wang X, Zhu J et al. Electrolyte design enables stable and energy-dense potassium-ion batteries. Angew Chem Int Ed 2025; 64: e202415491. 10.1002/anie.202415491 39387157

[17] Lv J, Wang B, Hao J et al. Single-crystalline Mn-based oxide as a high-rate and long-life cathode material for potassium-ion battery. eScience 2023; 3: 100081. 10.1016/j.esci.2022.10.007

[18] Wang L, Zhu J, Li N et al. Superior electrochemical performance of alkali metal anodes enabled by milder Lewis acidity. Energy Environ Sci 2024; 17: 3470–81. 10.1039/D4EE00900B

[19] Goubard-Bretesche N, Kemnitz E, Pinna N. A general low-temperature synthesis route to polyanionic vanadium phosphate fluoride cathode materials: AVPO_4_F (A = Li, Na, K) and Na_3_V_2_(PO_4_)_2_F_3_. Mater Chem Front 2019; 3: 2164–74. 10.1039/C9QM00325H

[20] Park H, Ko W, Lee Y et al. K_1.5_VOPO_4_F_0.5_: A novel high-power and high-voltage cathode for rechargeable K-ion batteries. J Mater Chem A 2021; 9: 11802–11. 10.1039/D1TA02247D

[21] Kim M, Lee S, Kang B. Fast-rate capable electrode material with higher energy density than LiFePO_4_: 4.2 V LiVPO_4_F synthesized by scalable single-step solid-state reaction. Adv Sci 2016; 3: 1500366. 10.1002/advs.201500366 PMC5064735 27774395

[22] Liu ZM, Wang J, Lu BA. Plum pudding model inspired KVPO_4_F@3DC as high-voltage and hyperstable cathode for potassium ion batteries. Sci Bull 2020; 65: 1242–51. 10.1016/j.scib.2020.04.010 36747411

[23] Xie C, Liu XW, Han J et al. Pomegranate-like KVPO_4_F@C microspheres as high-volumetric-energy-density cathode for potassium-ion batteries. Small 2022; 18: 2204348. 10.1002/smll.202204348 36336632

[24] He XD, Zhang LM, Jiang CH et al. Elevating cyclability of an advanced KVPO_4_F cathode via multi-component coating strategy for high-performance potassium-ion batteries. Chem Eng J 2022; 433: 134634. 10.1016/j.cej.2022.134634

[25] Liao JY, Hu Q, He XD et al. A long lifespan potassium-ion full battery based on KVPO_4_F cathode and VPO_4_ anode. J Power Sources 2020; 451: 227739. 10.1016/j.jpowsour.2020.227739

[26] Sui YL, Wu L, Hong W et al. Synthesis and electrochemical properties of spherically shaped LiVPO_4_F/C cathode material by a spray drying-roasting method. Rare Met 2021; 40: 72–7. 10.1007/s12598-019-01340-0

[27] Ellis DA, Mabury SA, Martin JW et al. Thermolysis of fluoropolymers as a potential source of halogenated organic acids in the environment. Nature 2001; 412: 321–4. 10.1038/35085548 11460160

[28] Conesa JA, Font R. Polytetrafluoroethylene decomposition in air and nitrogen. Polym Eng Sci 2001; 41: 2137–47. 10.1002/pen.10908

[29] Simon C, Kaminsky W. Chemical recycling of polytetrafluoroethylene by pyrolysis. Polym Degrad Stab 1998; 62: 1–7. 10.1016/S0141-3910(97)00097-9

[30] Dolui S, Kumar D, Banerjee S et al. Well-defined fluorinated copolymers: current status and future perspectives. Acc Mater Res 2021; 2: 242–51. 10.1021/accountsmr.1c00015

[31] Wernert R, Iadecola A, Stievano L et al. Origin of vanadium site sequential oxidation in K*_x_*VPO_4_F_1-_*_y_*O*_y_*. Chem Mater 2023; 35: 617–27. 10.1021/acs.chemmater.2c03132

[32] Wernert R, Nguyen LHB, Iadecola A et al. Self-discharge mechanism of high-voltage KVPO_4_F for K-ion batteries. ACS Appl Energy Mater 2022; 5: 14913–21. 10.1021/acsaem.2c02379

[33] Rahmatika AM, Toyoda Y, Nguyen TT et al. Cellulose nanofiber and magnetic nanoparticles as building blocks constructing biomass-based porous structured particles and their protein adsorption performance. ACS Sustain Chem Eng 2020; 8: 18686–95. 10.1021/acssuschemeng.0c07542

[34] Liu Z, Liu X, Wang B et al. Ultra-thick, dense dual-encapsulated Sb anode architecture with conductively elastic networks promises potassium-ion batteries with high areal and volumetric capacities. eScience 2023; 3: 100177. 10.1016/j.esci.2023.100177

[35] Möncke D, Eckert H. Review on the structural analysis of fluoride-phosphate and fluoro-phosphate glasses. J Non-Cryst Solids 2019; 3: 100026.

[36] Jang K, Yoon H, Hyoung JS et al. Enhancement of hydrogen evolution activity by tailoring the electronic structure in ruthenium-heteroatom-doped cobalt iron phosphide nanoframes. Appl Catal B-Environ Energy 2024; 341: 123327. 10.1016/j.apcatb.2023.123327

[37] Xiao YB, Ji Y, Ye YC et al. Enhancing the structure and properties of fluoro-sulfo-phosphate laser glass via Zn(PO_3_)_2_ incorporation. J Non-Cryst Solids 2021; 571: 121074. 10.1016/j.jnoncrysol.2021.121074

[38] Vollhardt KPC, Schore NE. Organic Chemistry. New York: WH Freeman, 1987.

[39] Illuminati G . Nucleophilic Heteroaromatic Substitution. Amsterdam: Elsevier, 1964, 285–371.

[40] Ouellette RJ, Rawn JD. Organic Chemistry Study guide: Key concepts, problems, and Solutions. Amsterdam: Elsevier, 2014.

[41] Hamlin TA, Swart M, Bickelhaupt FM. Nucleophilic substitution (S_N_2): dependence on nucleophile, leaving group, central atom, substituents, and solvent. ChemPhysChem 2018; 19: 1315–30. 10.1002/cphc.201701363 29542853 PMC6001448

[42] Lian RQ, Wang DS, Ming X et al. Phase transformation, ionic diffusion, and charge transfer mechanisms of KVOPO_4_ in potassium ion batteries: first-principles calculations. J Mater Chem A 2018; 6: 16228–34. 10.1039/C8TA06708B

[43] Heng YL, Gu ZY, Guo JZ et al. Low-strain and high-energy KVPO_4_F cathode with multifunctional stabilizer for advanced potassium-ion batteries. Energy Environ Mater 2024; 7: e12721. 10.1002/eem2.12721

[44] Chen XJ, Xia Y, Fang XP et al. Fe-substituted Mn-based Prussian white as cathode for high-performance potassium-ion battery. J Mater Sci 2022; 57: 14015–25. 10.1007/s10853-022-07430-2

[45] Deng T, Fan XL, Chen J et al. Layered P2-type K_0.65_Fe_0.5_Mn_0.5_O_2_ microspheres as superior cathode for high-energy potassium-ion batteries. Adv Funct Mater 2018; 28: 1800219. 10.1002/adfm.201800219

[46] Zhang XY, Yang YB, Qu XL et al. Layered P2-type K_0.44_Ni_0.22_Mn_0.78_O_2_ as a high-performance cathode for potassium-ion batteries. Adv Funct Mater 2019; 29: 1905679. 10.1002/adfm.201905679

[47] Chen F, Liao JY, Wang JR et al. Introducing a cell moisturizer: organogel nano-beads with rapid response to electrolytes for Prussian white analogue based non-aqueous potassium ion battery. Chem Commun 2020; 56: 9719–22. 10.1039/D0CC03646C 32815959

[48] Lu Y, Zhao CZ, Huang JQ et al. The timescale identification decoupling complicated kinetic processes in lithium batteries. Joule 2022; 6: 1172–98. 10.1016/j.joule.2022.05.005

[49] Yang Z, Wang B, Chen Y et al. Activating sulfur oxidation reaction via six-electron redox mesocrystal NiS_2_ for sulfur-based aqueous batteries. Natl Sci Rev 2023; 10: nwac268. 10.1093/nsr/nwac268 37181097 PMC10171633

[50] Zhou W, Fan HJ, Zhao D et al. Cathodic electrolyte engineering toward durable Zn–Mn aqueous batteries. Natl Sci Rev 2023; 10: nwad265. 10.1093/nsr/nwad265 37954197 PMC10632780

[51] Cai M, Zhang H, Zhang Y et al. Boosting the potassium-ion storage performance enabled by engineering of hierarchical MoSSe nanosheets modified with carbon on porous carbon sphere. Sci Bull 2022; 67: 933–45. 10.1016/j.scib.2022.02.007 36546028

[52] Xiao B, Sun Z, Zhang H et al. Enabling highly-efficient and stable potassium-ion storage by exposing atomic-dispersed super-coordinated antimony O_2_Sb_1_N_4_ sites on N-doped carbon nanosheets. Energy Environ Sci 2023; 16: 2153–66. 10.1039/D2EE03970B

[53] Qiu DP, Hou YL. Carbon materials toward efficient potassium storage: rational design, performance evaluation and potassium storage mechanism. Green Energy Environ 2023; 8: 115–40. 10.1016/j.gee.2022.05.007

[54] Yang H, He F, Liu F et al. Simultaneous catalytic acceleration of white phosphorus polymerization and red phosphorus potassiation for high-performance potassium-ion batteries. Adv Mater 2024; 36: 2306512. 10.1002/adma.202306512 37837252

[55] Jain R, Lakhnot AS, Bhimani K et al. Nanostructuring versus microstructuring in battery electrodes. Nat Rev Mater 2022; 7: 736–46. 10.1038/s41578-022-00454-9

[56] Meng ZF, Ma XT, Azhari L et al. Morphology controlled performance of ternary layered oxide cathodes. Commun Mater 2023; 4: 90. 10.1038/s43246-023-00418-8

[57] Li Q, Liu X, Tao Y et al. Sieving carbons promise practical anodes with extensible low-potential plateaus for sodium batteries. Natl Sci Rev 2022; 9: nwac084. 10.1093/nsr/nwac084 35992230 PMC9385462

[58] Qiu DP, Zhang B, Zhang T et al. Sulfur-doped carbon for potassium-ion battery anode: insight into the doping and potassium storage mechanism of sulfur. ACS Nano 2022; 16: 21443–51. 10.1021/acsnano.2c09845 36484831

[59] Sandler JKW, Kirk JE, Kinloch IA et al. Ultra-low electrical percolation threshold in carbon-nanotube-epoxy composites. Polymer 2003; 44: 5893–9. 10.1016/S0032-3861(03)00539-1

[60] Entwistle J, Ge RH, Pardikar K et al. Carbon binder domain networks and electrical conductivity in lithium-ion battery electrodes: a critical review. Renew Sust Energ Rev 2022; 166: 112624. 10.1016/j.rser.2022.112624

[61] Li J, Ma PC, Chow WS et al. Correlations between percolation threshold, dispersion state, and aspect ratio of carbon nanotubes. Adv Funct Mater 2007; 17: 3207–15. 10.1002/adfm.200700065

[62] Chen F, Han J, Kong D et al. 1000 Wh L^−1^ lithium-ion batteries enabled by crosslink-shrunk tough carbon encapsulated silicon microparticle anodes. Natl Sci Rev 2021; 8: nwab012. 10.1093/nsr/nwab012 34691733 PMC8433081

