# Supplementary material for: Nucleophilic cleavage of C–F bonds by Brønsted base for rapid synthesis of fluorophosphate materials
Source: Natl Sci Rev. 2025 Jan 21;12(3):nwaf020. doi: 10.1093/nsr/nwaf020 (PMC11841365; doi:10.1093/nsr/nwaf020)
Supplement: nwaf020_Supplemental_File [file nwaf020_supplemental_file.pdf]

Supplementary Information for

Nucleophilic cleavage of C–F bonds by Brønsted base for rapid synthesis of fluorophosphate materials

Qingfeng Fu, Zihao Chang, Peng Gao, Wang Zhou, Hongliang Dong, Peifeng Huang, Aiping Hu, Changling Fan, Peitao Xiao, Yufang Chen,* Jilei Liu,*

Corresponding author: *chenyufang@nudt.edu.cn (Yufang Chen); liujilei@hnu.edu.cn (Jilei Liu)*

The PDF file includes:

Supplementary Text
Figs. S1 to S42
Table S1
References

MATERIALS AND METHODS

MATERIALS SYNTHESIS

Synthesis of KVPF-P&P

The pure phase $KVPO_4F$ porous microsphere (designated as KVPF-P&P) was prepared by using a novel, straightforward and scalable spray-dry process (**Fig. S5a**). Firstly, 0.2 mol oxalic acid dihydrate and 0.2 mol V_2O_5 were dissolved in 100 mL H_2O deionized water and stirred for 30 min at 80 ℃. After a blue solution was formed, KF (Aladdin, 99%) and $NH_4H_2PO_4$ (Aladdin, 99%) with stoichiometric ratio were added to solution with vigorous stirring to form solution A. Concurrently, solution B containing 0.3g PVP (average M_w = 1300000) and 2g PTFE emulsion dissolved in 30 mL deionized water was prepared. Then, solution B was then slowly added to solution A with continuous stirring to form the feed solution. Finally, the feed solution was spray-dry at 180 ℃ to obtain a green powder. The obtained product was stabilized at 400 ℃ for 6 hours and further calcinated at 750 ℃ for 3 hours under an Ar atmosphere to yield the final KVPF-P&P compounds.

Synthesis of KVPF-No PTFE

For comparison, KVPF-No PTFE was also prepared in the same way. The synthesis process of KVPF-No PTFE is the same as that of KVPF-P&P, except that the solution B is changed. Solution B was prepared by dissolving 0.3g PVP (average M_w = 1300000) in 30 mL distilled water (**Fig. S5b**).

Synthesis of KVPF-No P&P

For comparison, KVPF-No P&P was prepared in the same way. The synthesis process of KVPF-No PTFE is the same as that of KVPF-P&P, except that the configuration of solution B is not performed (**Fig. S5c**).

Synthesis of KVPF-CTR

For comparison, the conventional two-step carbothermal reduction (CTR) method was used to obtain $KVPO_4F$ powder (designated as KVPF-CTR) according to the method described elsewhere [1], which included the preparation of VPO_4 as the first step and subsequent heat-treatment with KF at 750 ℃ for 3 hours in a tube furnace under Ar atmosphere (**Fig. S2**).

Synthesis of $LiVPO_4F$

The synthesis process of $LiVPO_4F$ is the same as that of KVPF-P&P, except that the compositions of the starting materials are changed (**Fig. S27a**).

Synthesis of $Na_3V_2(PO_4)_2F_3$

The synthesis process of $Na_3V_2(PO_4)_2F_3$ is the same as that of KVPF-P&P, except that the compositions of the starting materials are changed (**Fig. S27b**).

Cross-validation experiment

The cross-validation experiment setup as showed in **Fig. S26**. Typically, the commercial PTFE powder was loaded onto a quartz boat in the central heating zone and placed in the upstream zone. An as-prepared KVPF-No P＆P precursor was inverted on a square quartz boat in the downstream zone. The tube is then calcinated at 750 ℃ for 3 hours under an Ar.

MATERIALS CHARACTERIZATIONS

PXRD patterns were collected by a PANalytical Empyrean X-ray diffractometer with Cu Kα radiation. Rietveld refinements were performed by using the GSAS software + EXPGUI interface to reveal the lattice parameters. SEM (JSM-7610FPlus), STEM (Thermo Fisher Scientific microscope Themis Z 3.2), Brunauer–Emmett–Teller (BET, JW-BK200C, Beijing JWGB SCI & TECH) was used to measure the morphology. SEM measurements were conducted at KW-ST Lab (www.kewei-scitech.com). Raman were performed on WITEC alpha-300R equipped with 532 nm argon laser. FTIR were carried out using an Thermo Fisher Scientific Nicolet iS50. Thermal analysis was carried out with a TG-DSC STA 449 F5 Jupiter apparatus (Netzsch, Germany) combined with a mass spectrometer 204 F1 Phoenix (Netzsch, Germany). Carbon contents were measured with a combustion-IR Carbon/Sulfur determinator (LECO CS844). XAS measurements were performed on the beamline 1W1B and 4B9A at Beijing Synchrotron Radiation Facility (BSRF). The software package ATHENA was used for data analysis.

ELECTROCHEMICAL MEASUREMENTS

For the cathode preparation, slurry containing 80 wt% active material, 10 wt% polyvinylidene fluoride (PVDF, provided by Energy Chemical) and conductive carbon (CNT, provided by XFnano) was dissolved in N-methylpyrrolidinone (NMP, provided by Energy Chemical) solution. The resulting slurry was cast on aluminum current collector and dried at 120 ℃ overnight in vacuum oven. The electrochemical properties of $KVPO_4F$ were evaluated using CR2032-type coin cells (supplied by Guangdong Canrd Ltd.) with potassium metal foil as counter and reference electrodes and glass microfiber (Whatman) as the separator. The galvanostatic charge-discharge measurements were performed on a automatic battery testing system (Neware CT-4008T). In this work, 131 mA g^{-1} is equivalent to 1C, which is calculated based on the theoretical capacity of $KVPO_4F$ (131 mAh g^{-1}). The specific capacity is calculated based on the total mass of KVPF-P&P, which contains 4.44 wt% carbon, rather than the pure $KVPO_4F$. For the KVPF-P&P//graphite coin full-cell, the athode/anode area is 1.54 cm^2, the cathode active material loading mass is 2.5 mg, the negative-to-positive (N/P) ratio is 1.1. The 0.8 M KPF_6 in a mixture of 1:1 volume ratio ethylene carbonate (EC) and diethylene carbonate (DEC) with 1.5wt% Tris(trimethylsilyl) phosphine (provided by Adamas-beta) was used as electrolyte.

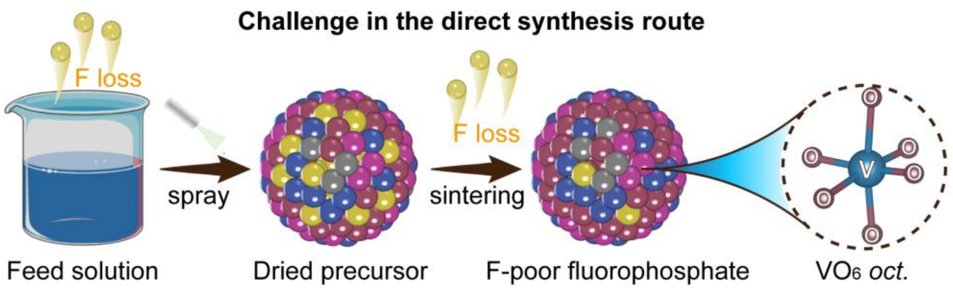

Fig. S1. Schematic illustration of the challenge in the direct synthesis route.

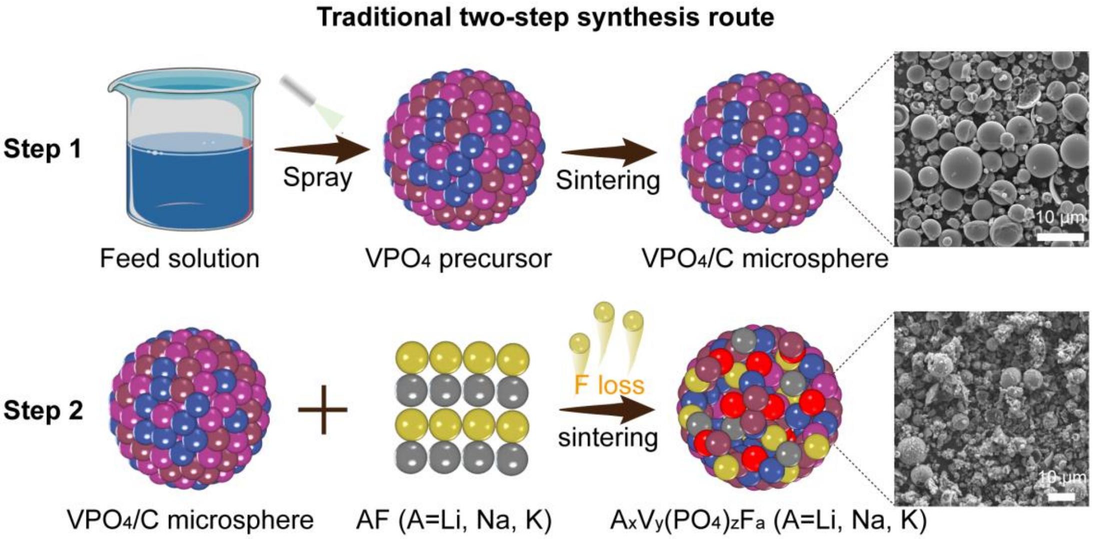

Fig. S2. Process for the preparation of $A_xV_y(PO_4)_zF_a$ (A=Li, Na, K) using a traditional two-step synthesis route.

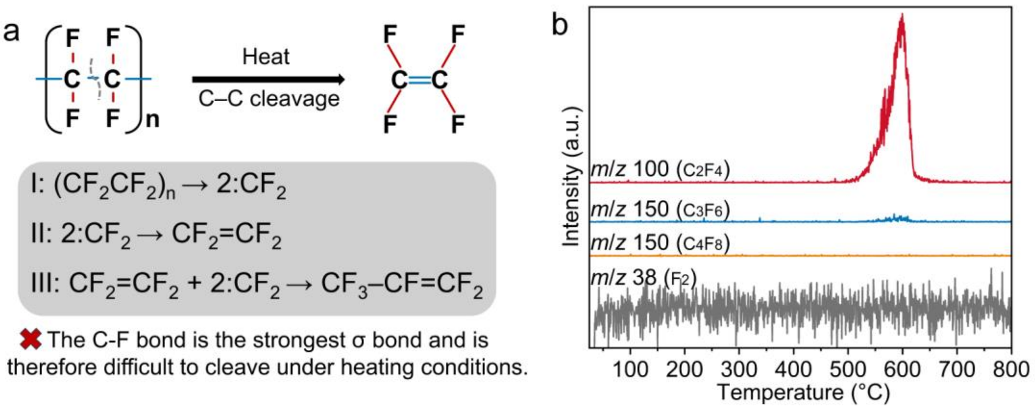

Fig. S3. The thermal decomposition path and its corresponding decomposition products of PTFE.

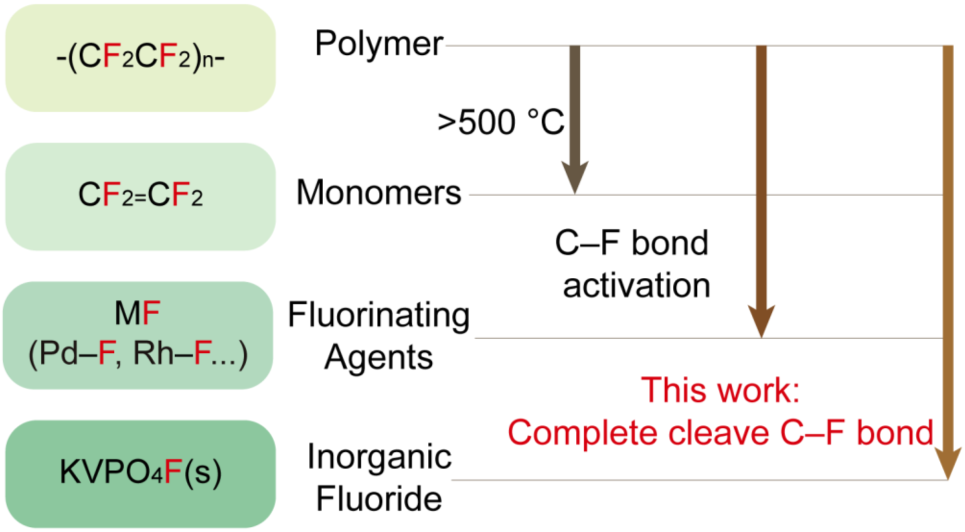

Fig. S4. The challenge and our strategies for cleaving the C–F bond.

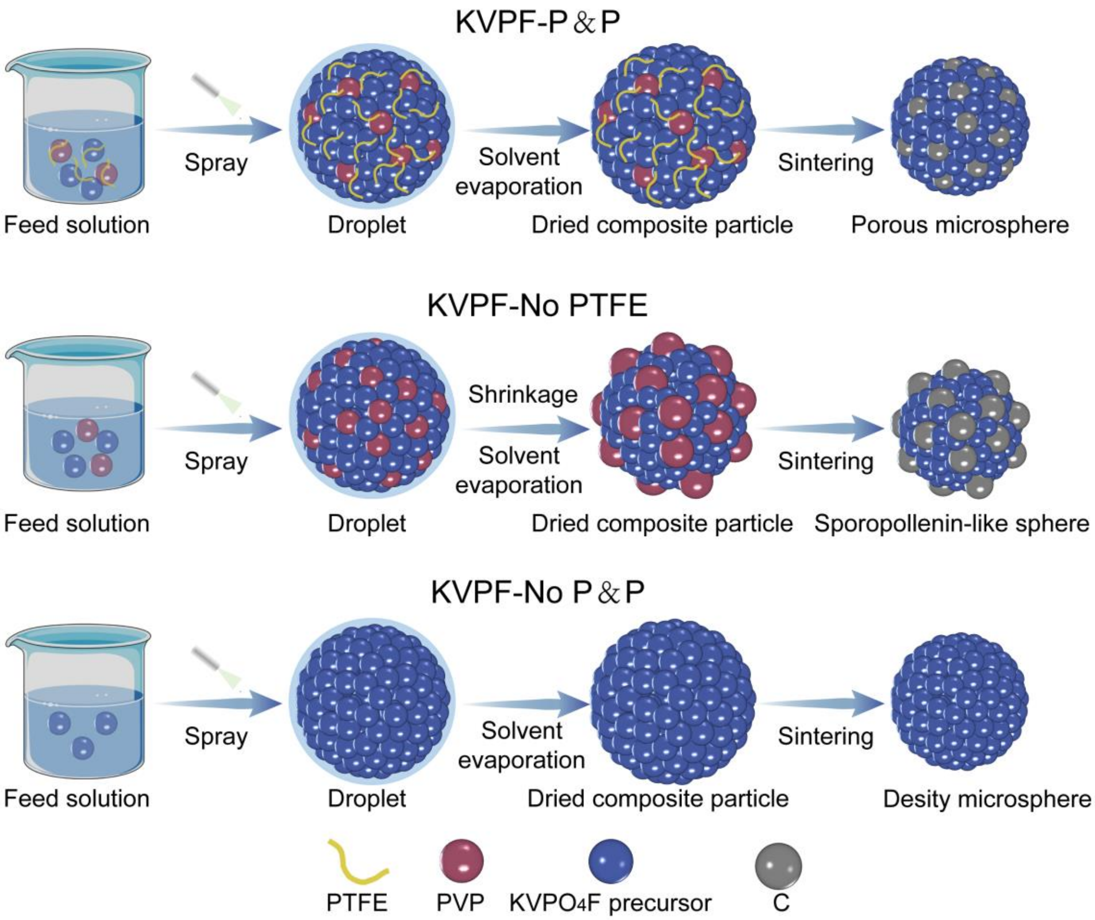

Fig. S5. Process for the preparation of KVPF-P&P, KVPF-No PTFE, and KVPF-No P&P.

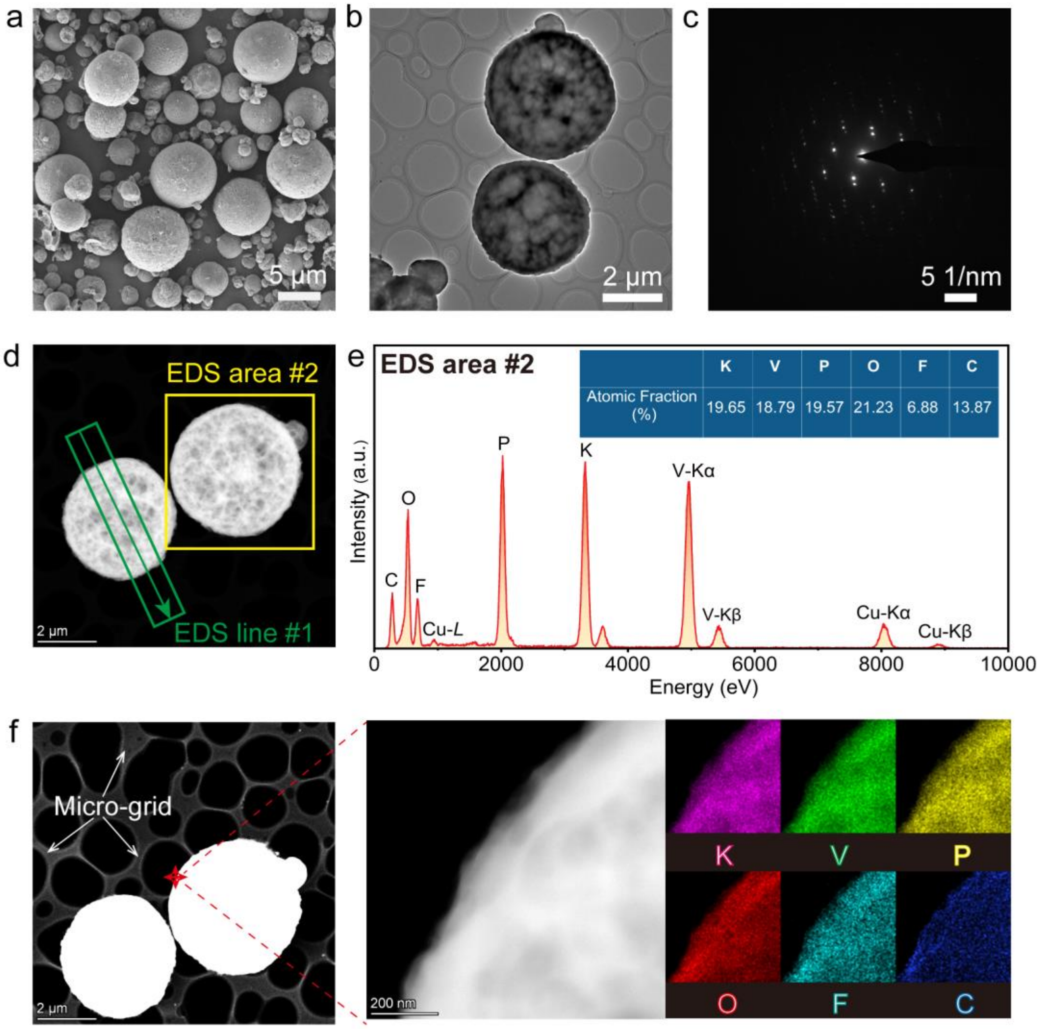

Fig. S6. (a) SEM and (b)TEM images, (c) SAED pattern, (d, e) STEM-EDX line scan and (f) STEM-EDX mapping of KVPF-P&P.

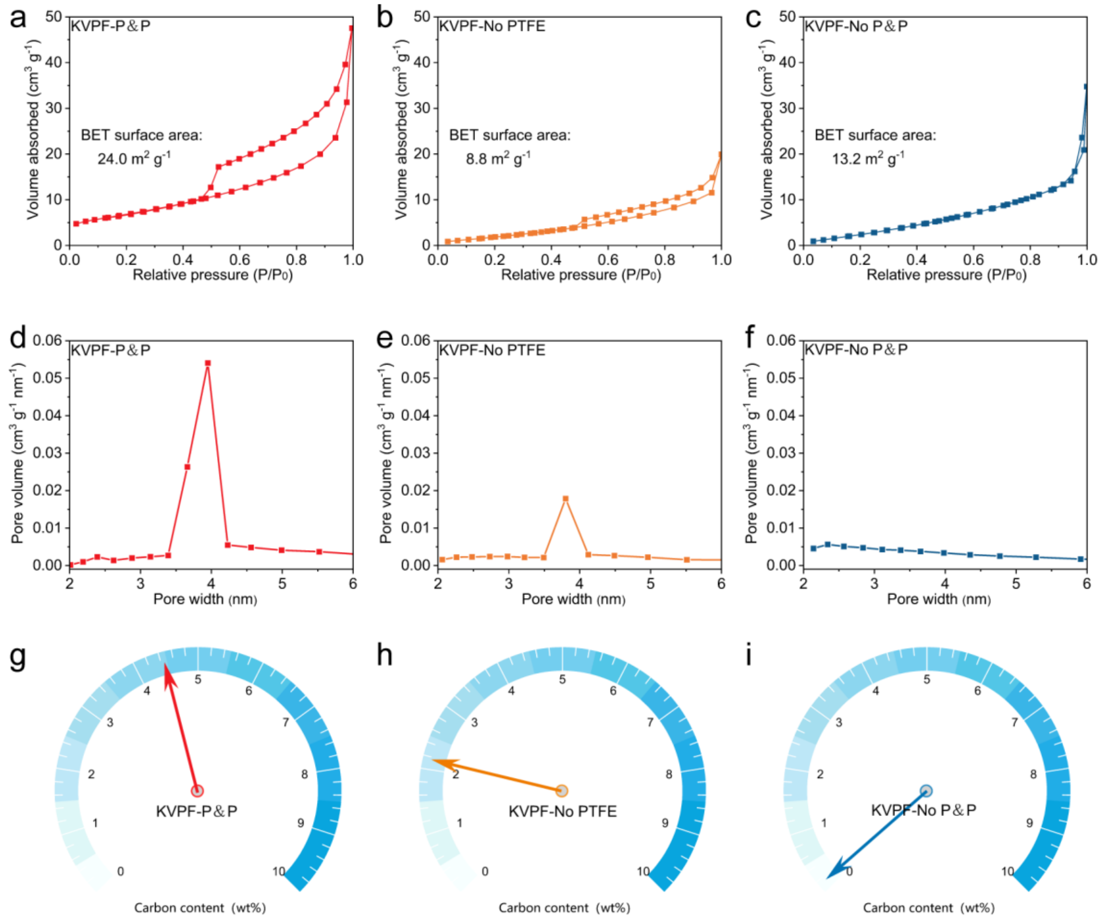

Fig. S7. N_2 adsorption-desorption isotherm and pore size distributions of (a, d) KVPF-P&P, (b, e) KVPF-No PTFE, and (c, f) KVPF-No P&P. Plots of carbon content of (g) KVPF-P&P, (h) KVPF-No PTFE, and (i) KVPF-No P&P.

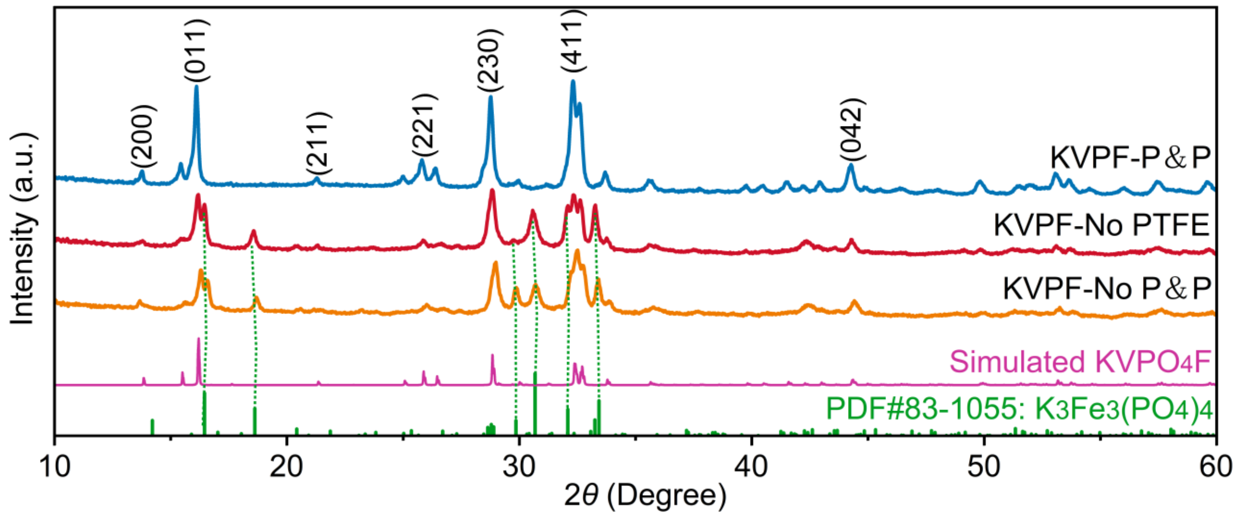

Fig. S8. PXRD patterns of KVPF-P&P, KVPF-No PTFE, and KVPF-No P&P.

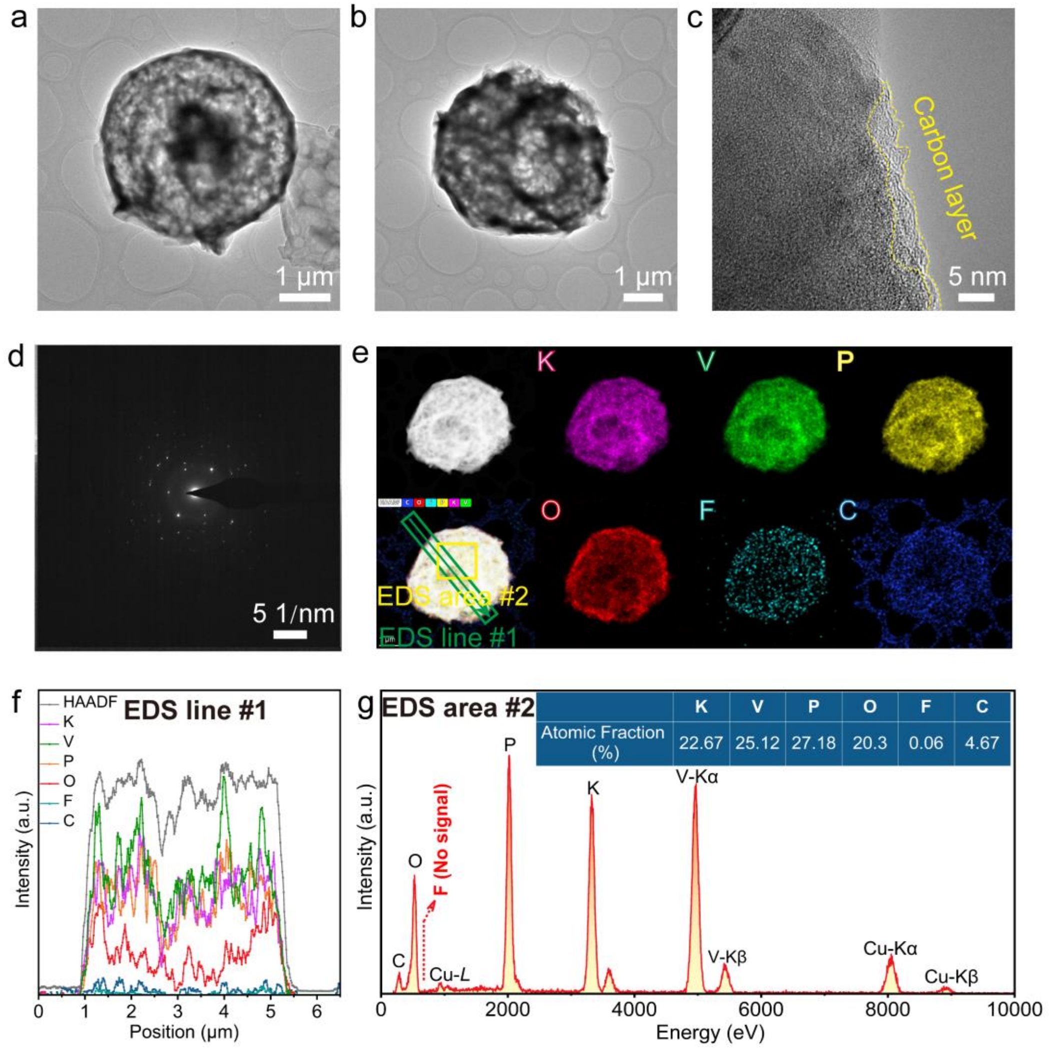

Fig. S9. (a, b) TEM images, (c) HRTEM pattern, (d) SAED, (e) STEM-EDX mapping and (f, g) STEM-EDX line scan of KVPF-No PTFE.

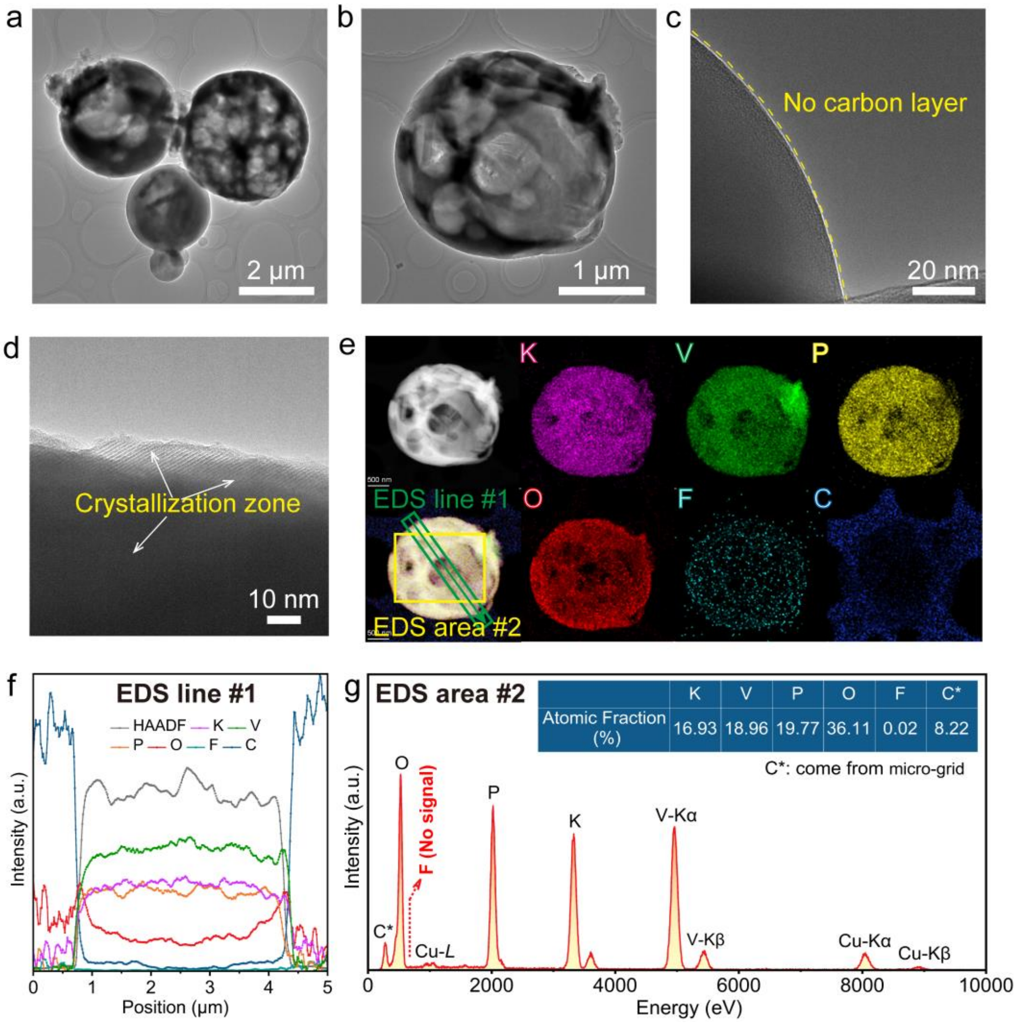

Fig. S10. (a, b, c) TEM images, (d) HRTEM pattern, (e) STEM-EDX mapping and (f, g) STEM-EDX line scan of KVPF-No P&P.

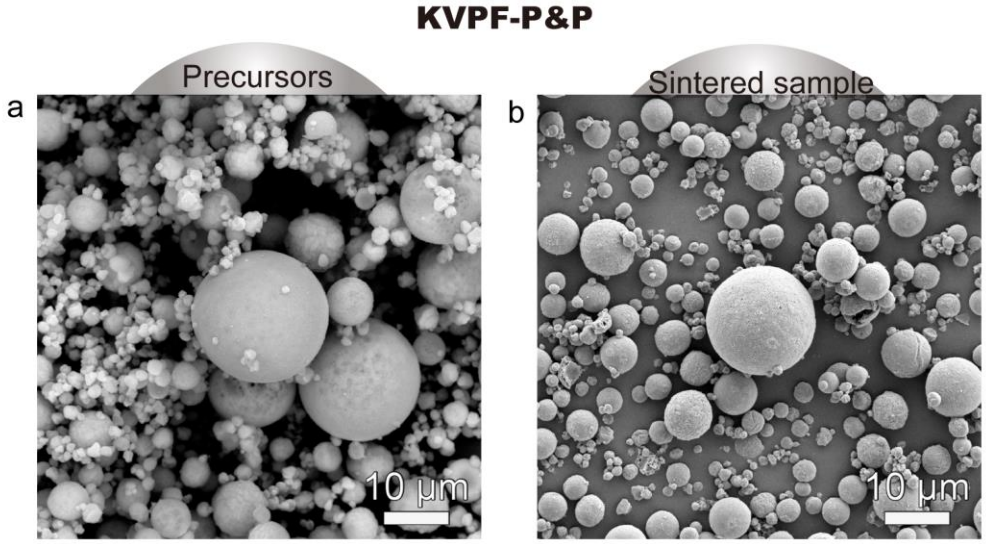

Fig. S11. SEM of (a) as-prepare KVPF-P&P precursor and (b) KVPF-P&P sample after sintered.

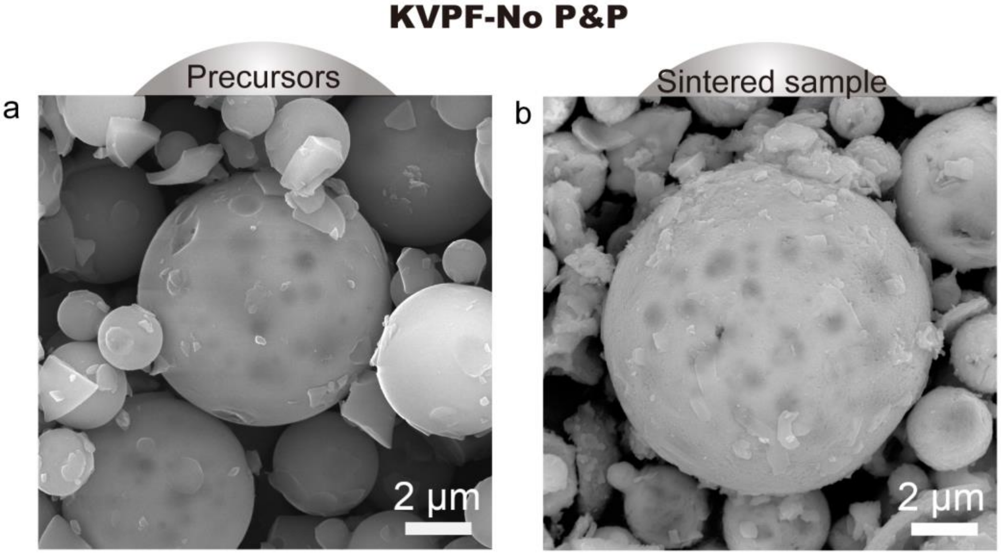

Fig. S12. SEM of (a) as-prepare KVPF-No P&P precursor and (b) KVPF-No P&P sample after sintered.

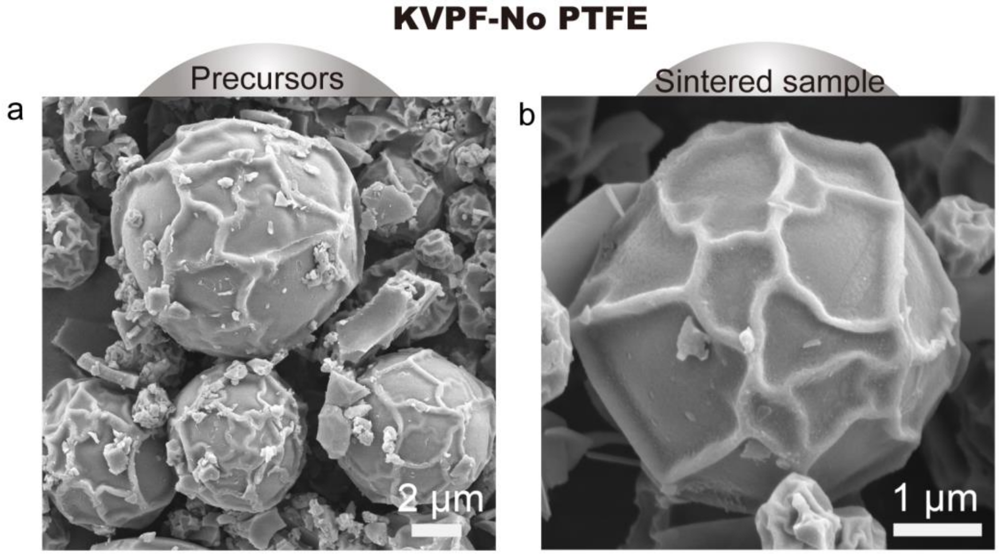

Fig. S13. SEM of (a) as-prepare KVPF-No PTFE precursor and (b) KVPF-No PTFE sample after sintered.

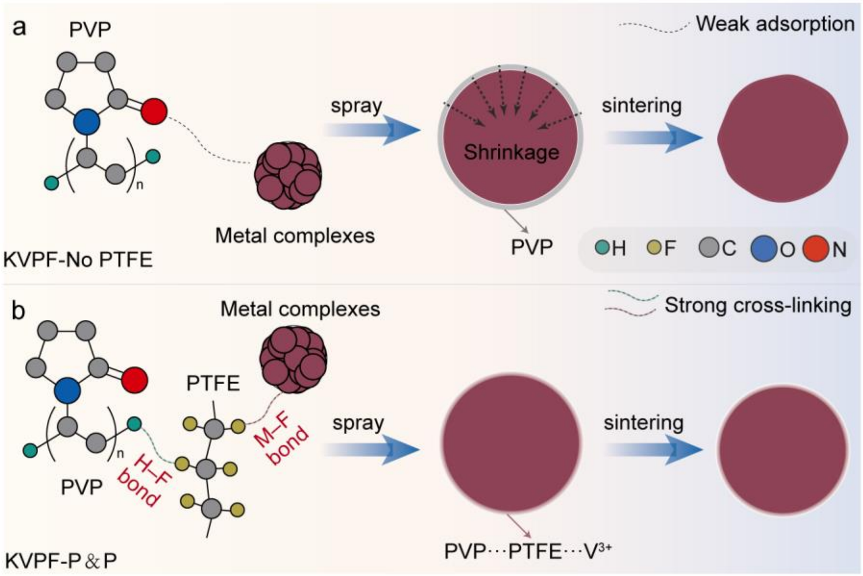

Fig. S14. The formation mechanism of (a) KVPT-No PTFE and (b) KVPF-P&P.

Notes: The volume shrinkage observed in KVPF-No PTFE is mainly due to the weak interactions between PVP and metal ions, resulting in irregularly thermal shrinking of PVP during the spray drying process. In contrast, although PVP is present in KVPF-P&P, its main function is to stabilize the PTFE emulsion through H–F bonding. Furthermore, the crosslinked PVP and PTFE could effectively disperse metal ions via M–F bonds, forming a robust interconnected network. This stable and rigid PVP ··PTFE ··V^{3+} chains hinder irregularly volumetric shrinkage of PVP during the spray drying process, and finally forms a porous microsphere structure [2-4].

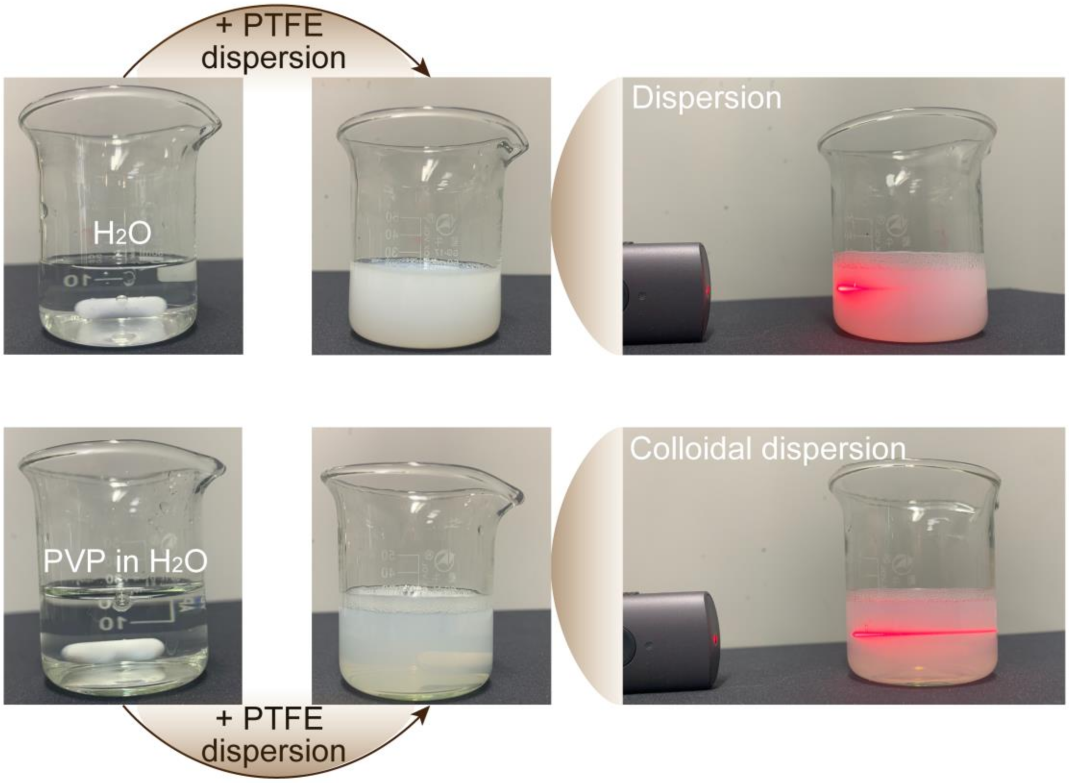

Fig. S15. Effect of PVP as surface dispersant on PTFE emulsion dispersion.

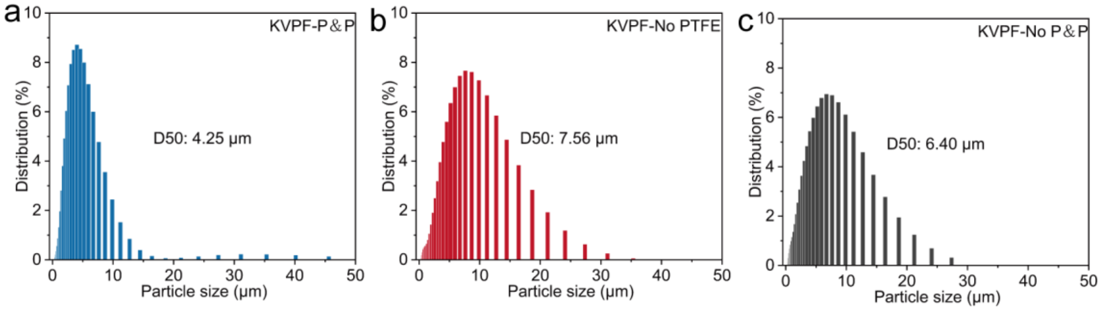

Fig. S16. Particle size distribution curves of (a) KVPF-P&P, (b) KVPT-No PTFE, and (c) KVPF-No P&P.

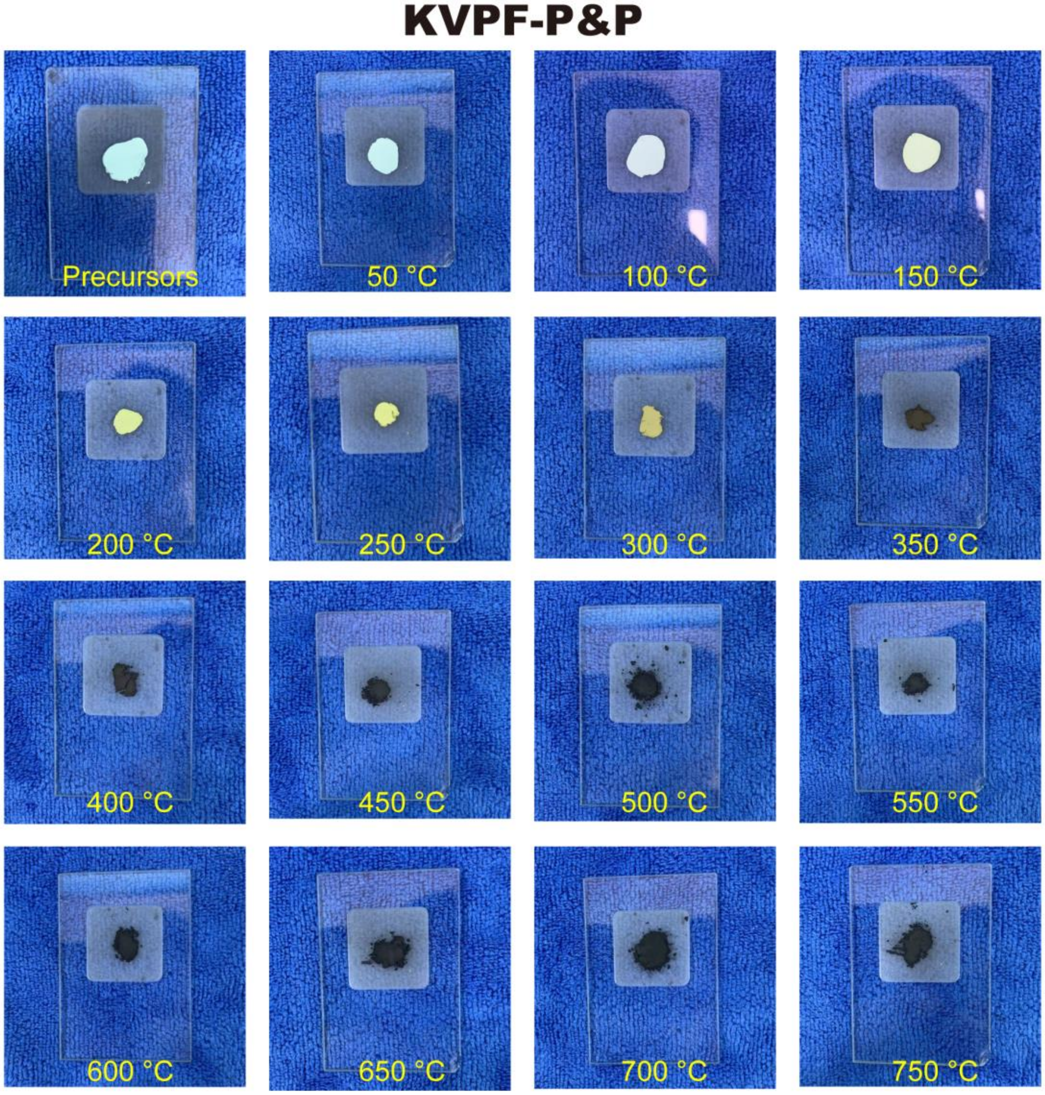

Fig. S17. Optical images at different calcination temperatures of KVPF-P&P.

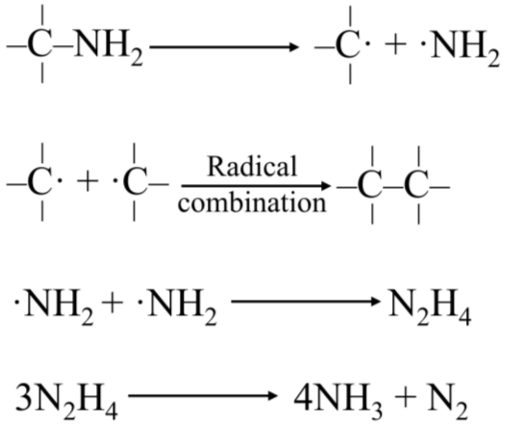

Fig. S18. The reaction of $R_3C–NH_3$ under high temperatures.

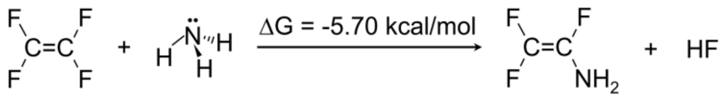

Fig. S19. Computed free energy profiles (in kcal/mol) at the B3LYP/Def2-SVP level of theory.

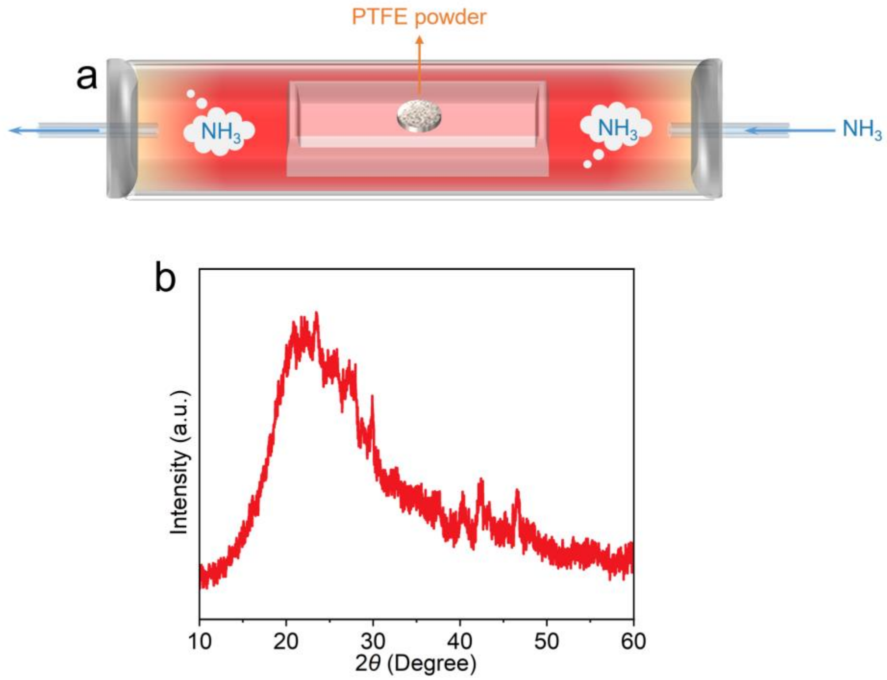

Fig. S20. (a) The validation experiment setup and (b) the PXRD of carbon product.

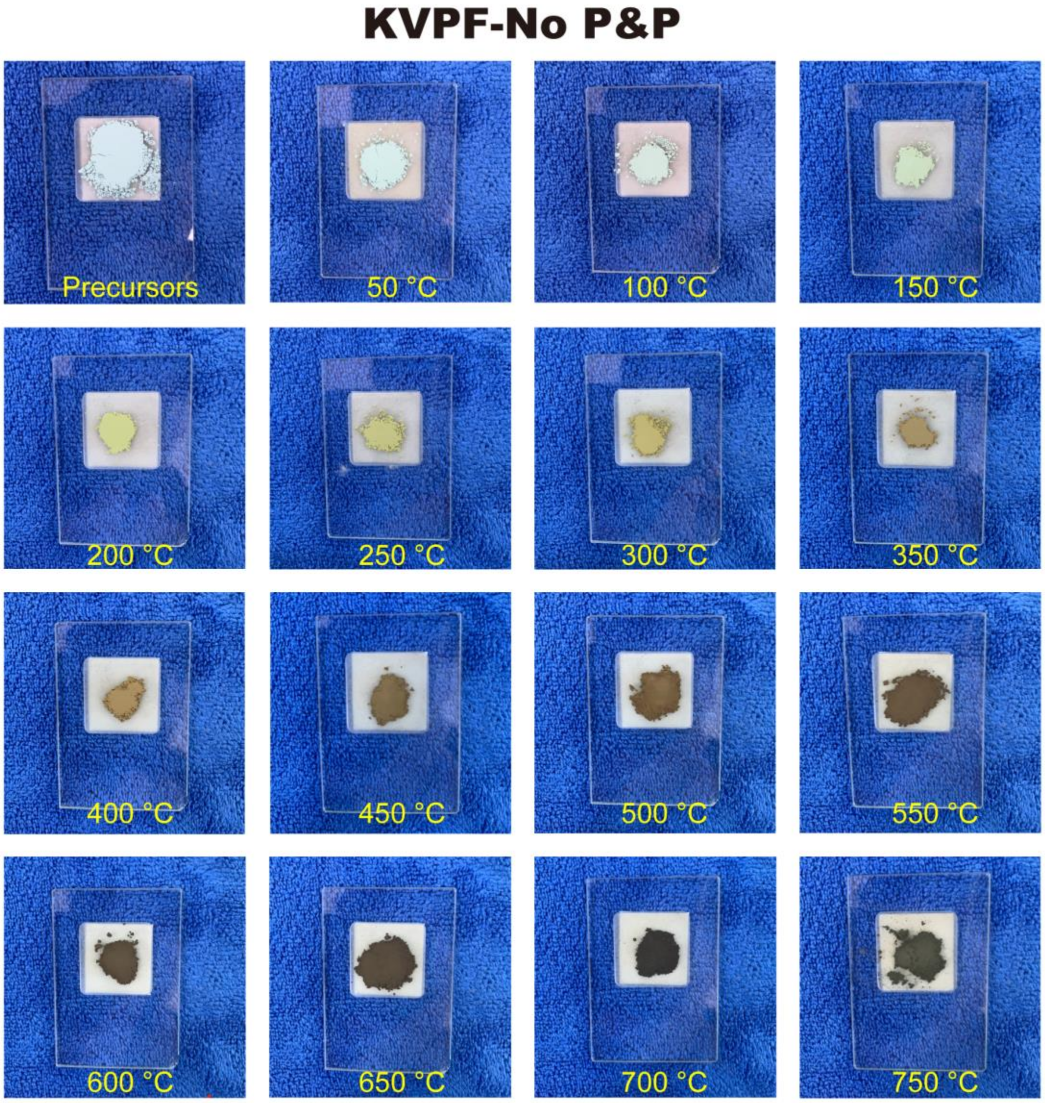

Fig. S21. Optical images at different calcination temperatures of KVPF-No P&P.

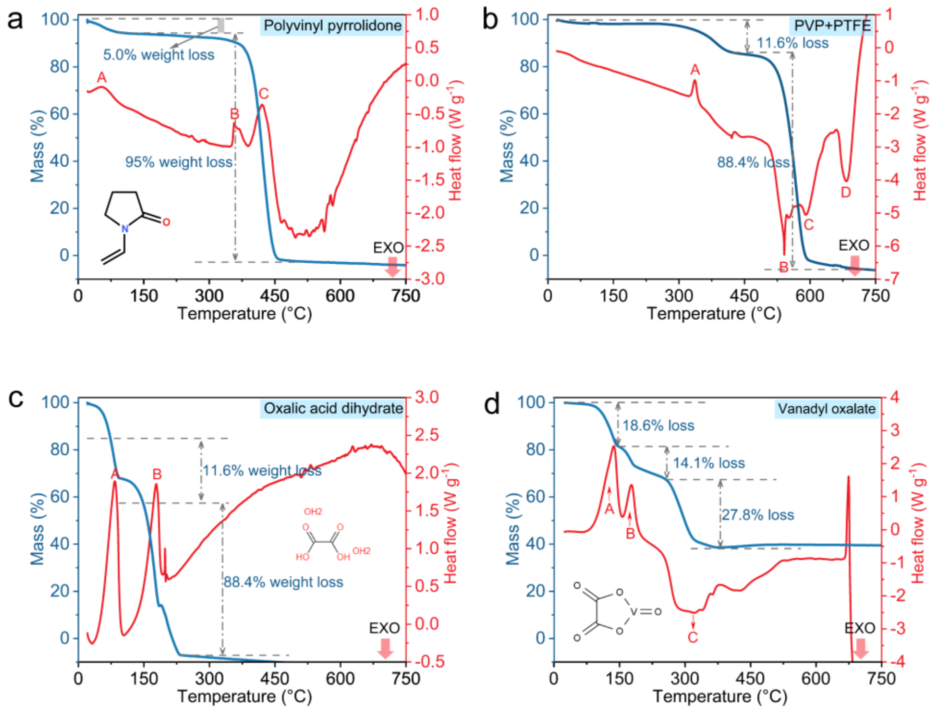

Fig. S22. TG/DSC curves of (a) PVP, (b) PVP+PTFE, (c) Oxalic acid dihydrate, and (d) Vanadyl oxalate.

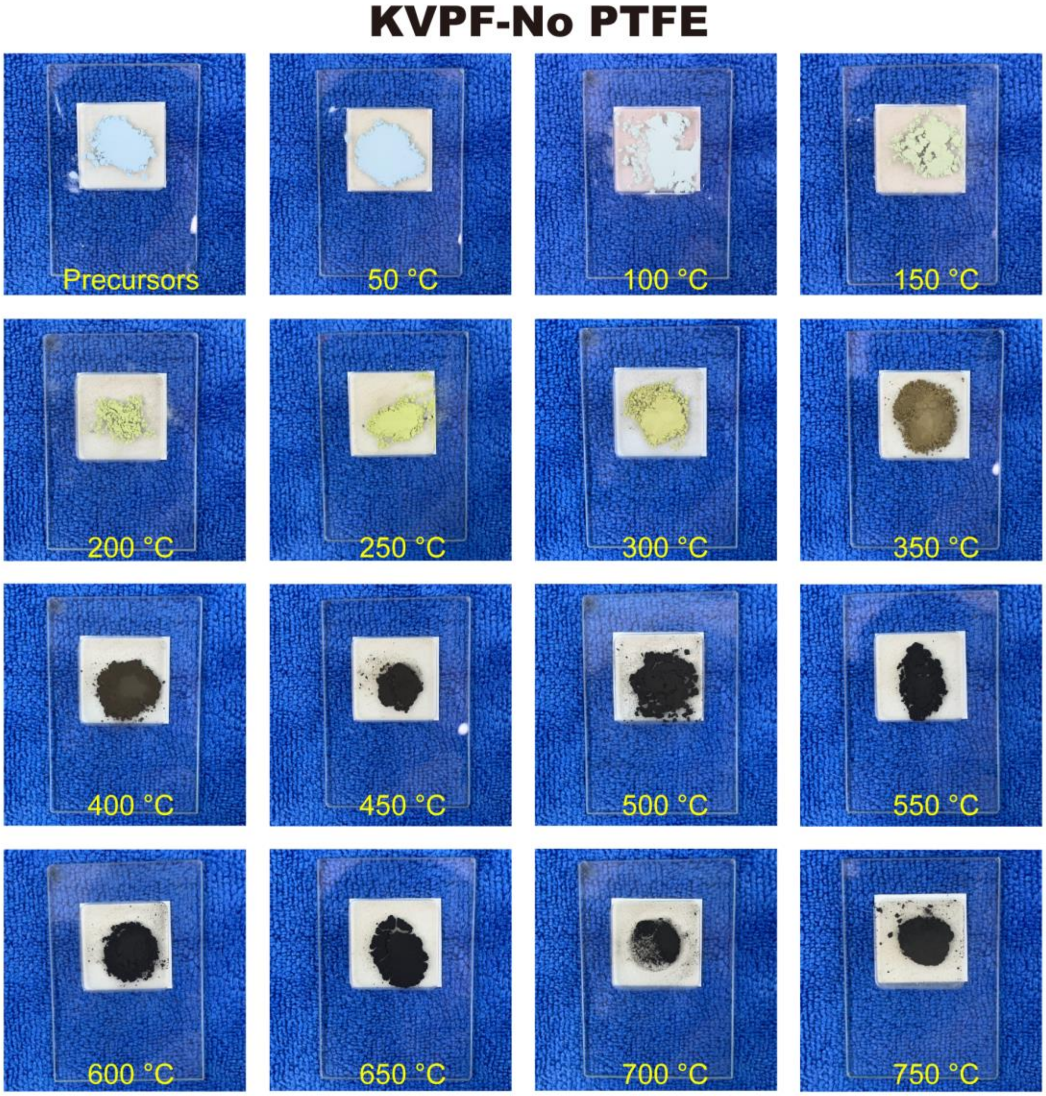

Fig. S23. Optical images at different calcination temperatures of KVPF-No PTFE.

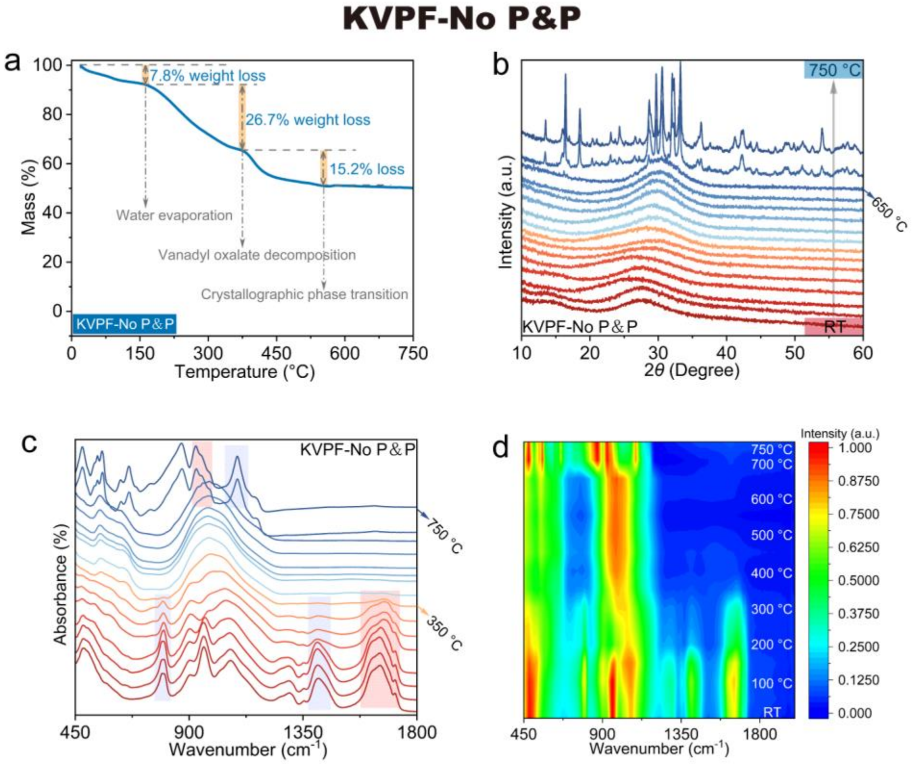

Fig. S24. (a) TG-DSC curves, (b) *Ex-situ* PXRD patterns and (c, d) *Ex-situ* FTIR spectra of the KVPF-No P&P precursor at different calcination temperatures.

Notes: TG curve analysis revealed three distinct weight losses at different temperature ranges in KVPF-No P&P precursor: 30–150 ℃ (~7.8% loss) corresponding to water evaporation, 150–350 ℃ (~26.7% loss) attributed to vanadyl oxalate decomposition, and 530–750 ℃ (~15.2% loss) indicating the formation of the final KVPF-PTFE product through crystallographic phase transition.

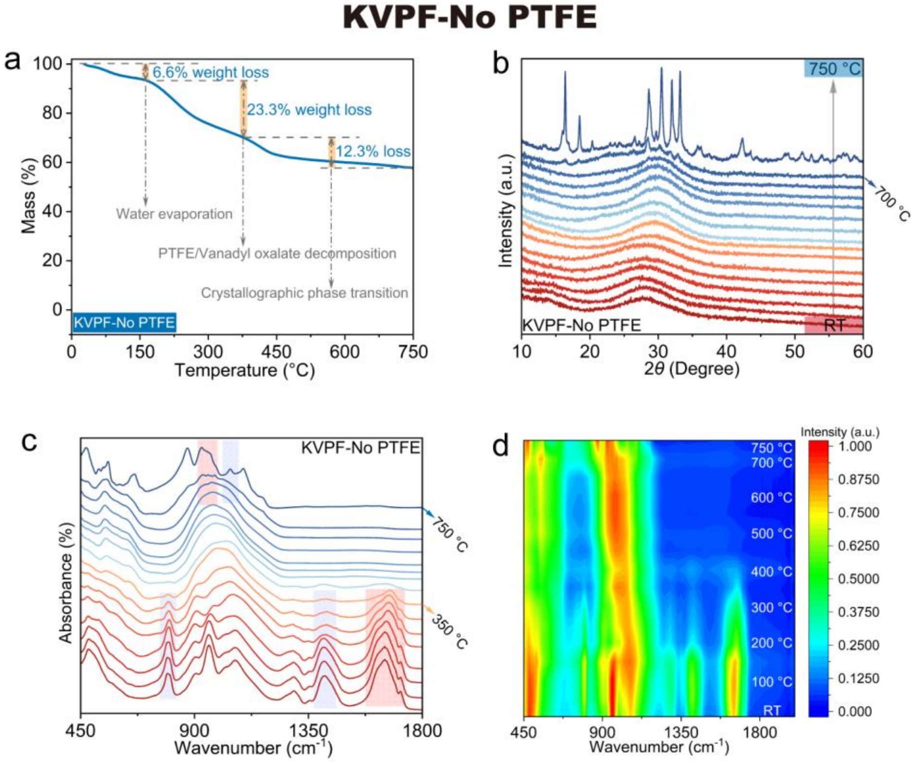

Fig. S25. (a) TG-DSC curves, (b) *Ex-situ* PXRD patterns and (c, d) *Ex-situ* FTIR spectra of the KVPF-No PTFE precursor at different calcination temperatures.

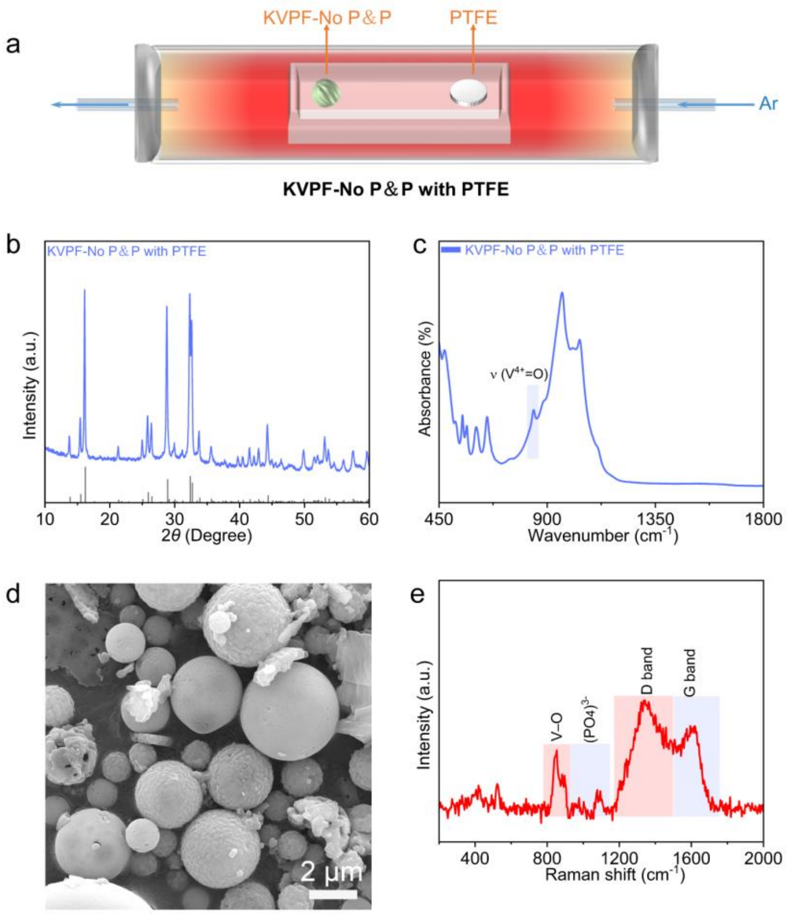

Fig. S26. (a) Schematic illustration the cross-validation experiment of KVPF-No P&P precursor by add commercial PTFE powder, (b) PXRD patterns, (c) FTIR spectra, SEM, and (d) Raman spectra.

Notes: Although the synthesis of KVPF-No P&P shows a $KTiOPO_4$-type structure in the cross-experiment, it still contains a small amount of V^{4+}=O peaks and irregular carbon distribution due to the fact that KVPF-No P&P was not mixed *in-situ* with PTFE.

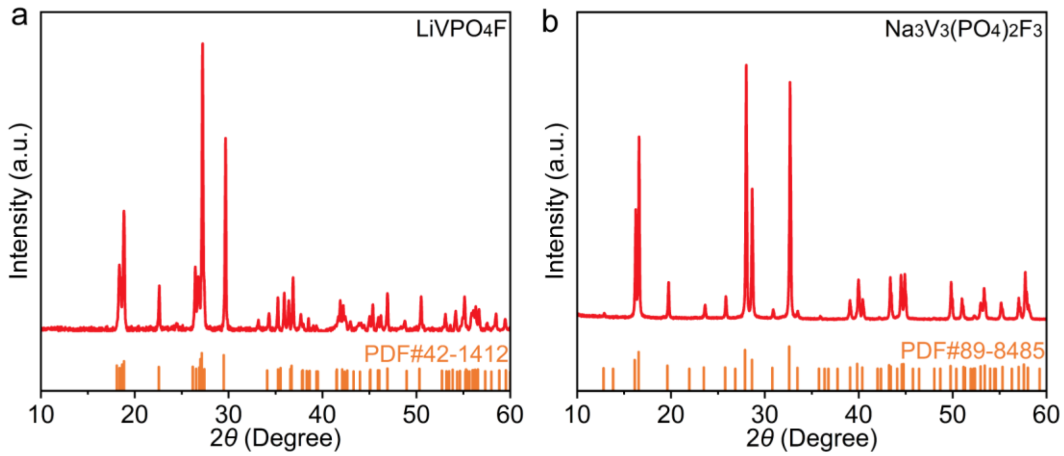

Fig. S27. PXRD patterns of (a) $LiVPO_4F$ and (b) $Na_3V_3(PO_4)_2F_3$.

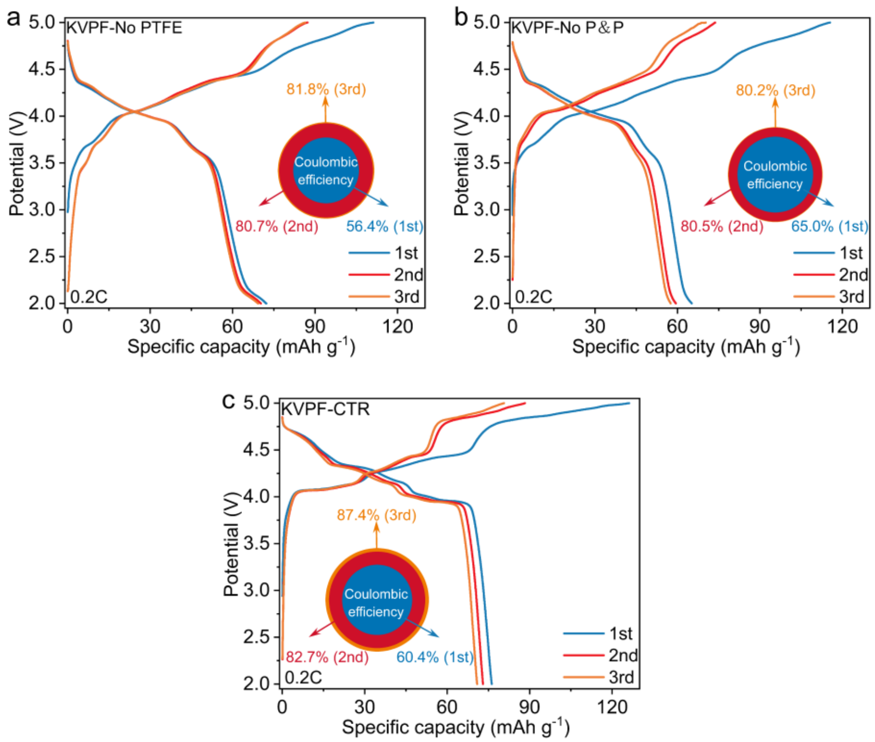

Fig. S28. Charging/discharging curves of (a) KVPF-No PTFE, (b) KVPF-No P＆P, and (c) KVPF-CTR at 0.2C.

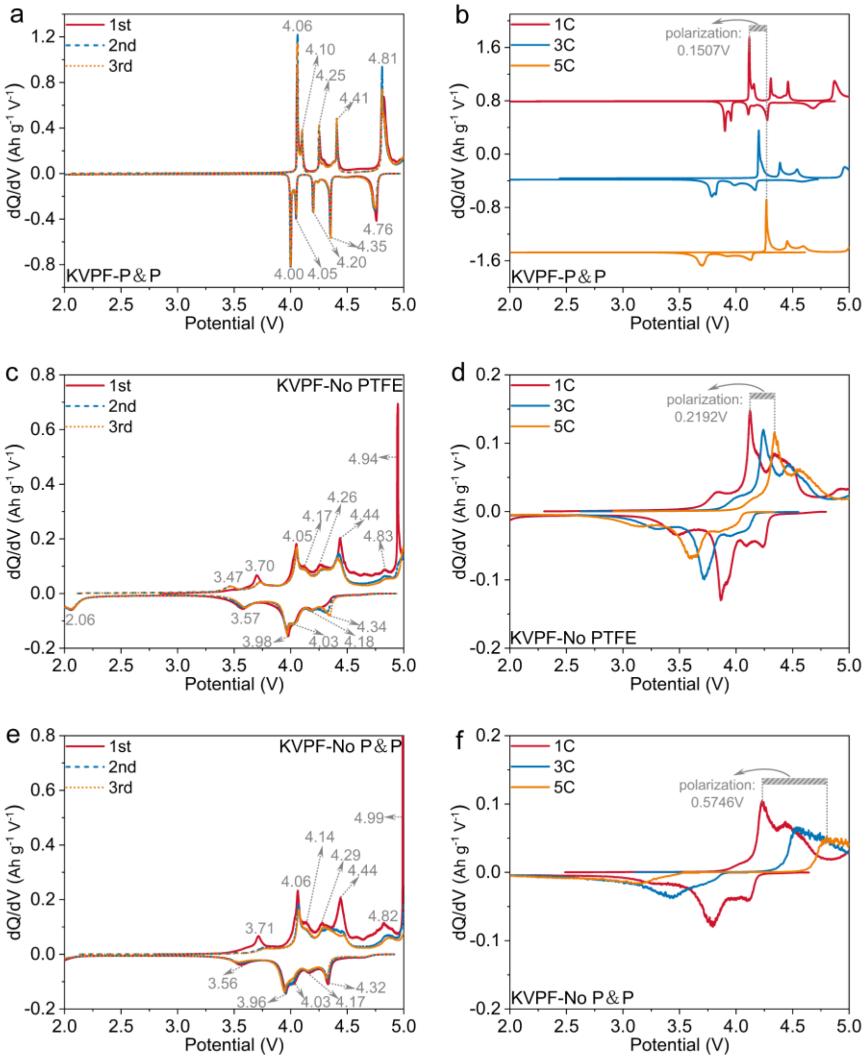

Fig. S29. CV curves of (a) KVPF-P&P, (c) KVPF-PTFE and (e) KVPF-No P＆P at 0.1 mV s^{-1}. CV curves of (b) KVPF-P&P, (d) KVPF-PTFE and (f) KVPF-No P＆P at selected scan rates.

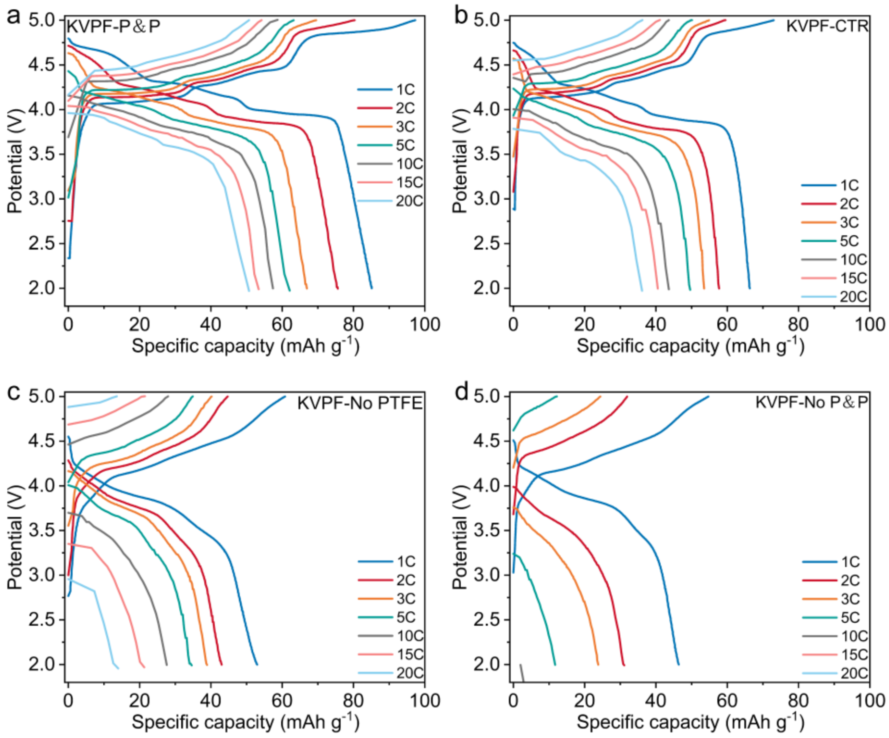

Fig. S30. Charging/discharging curves of (a) KVPF-P&P, (b) KVPF-CTR, (c) KVPF-No PTFE and (d) KVPF-No P&P at different current densities.

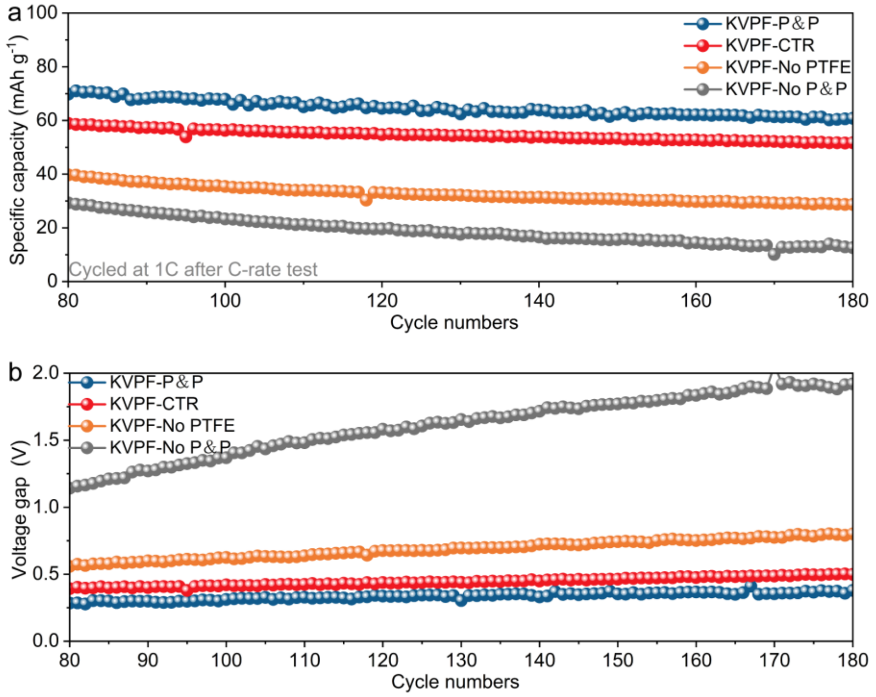

Fig. S31. (a) Long-term cyclability and (b) voltage gap of KVPF-P&P, (b) KVPF-CTR, (c) KVPF-No PTFE and (d) KVPF-No P&P at different current densities.

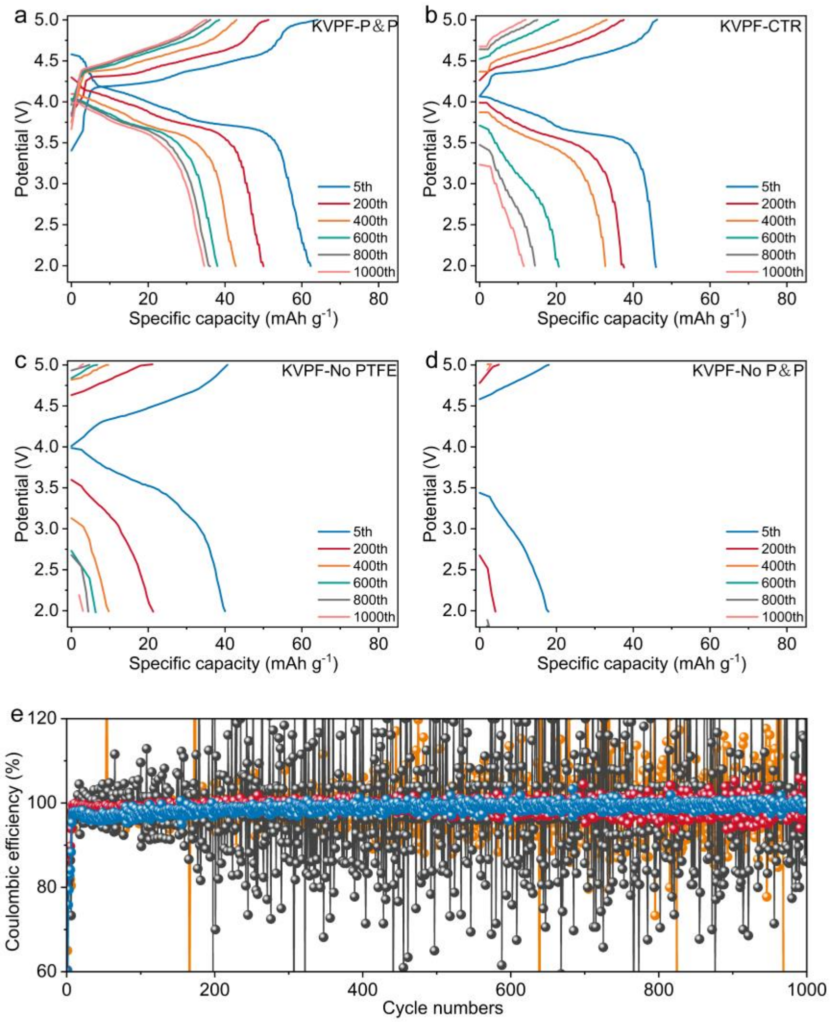

Fig. S32. (a, b, c, d) Cyclability curves and (e) Coulombic efficiency of KVPF-P&P, KVPF-CTR, KVPF-No PTFE and KVPF-No P＆P at 5C.

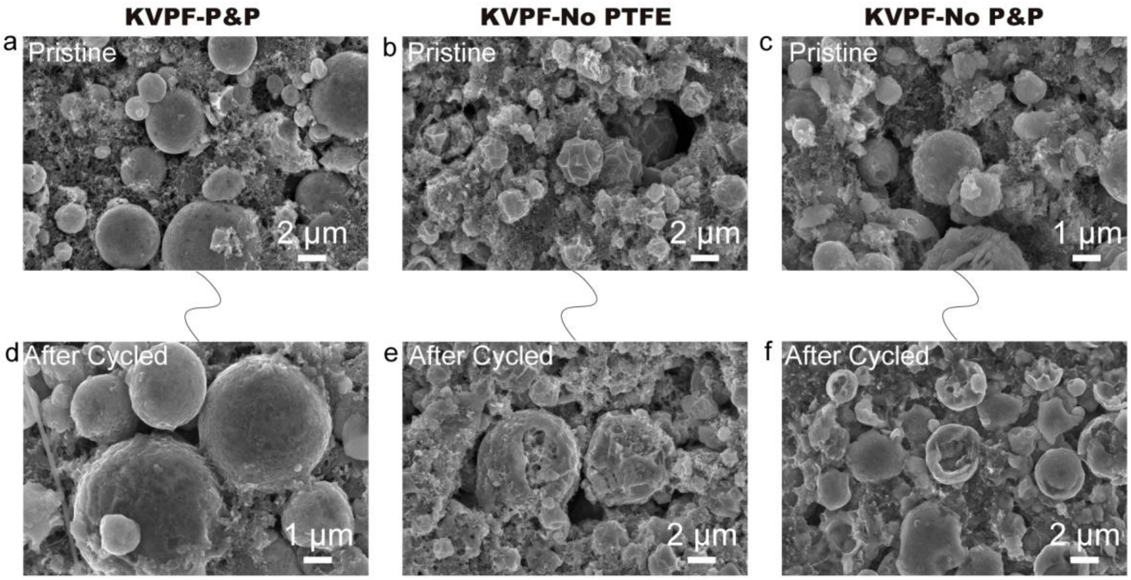

Fig. S33. *Ex-situ* SEM patterns of (a, d) KVPF-P&P, (b, e) KVPT-No PTFE and (c, f) KVPF-No P&P.

Notes: *Ex-situ* SEM analysis also confirms that the porous structure was preserved after 1000 cycles for KVPF-P&P, demonstrating its structural stability.

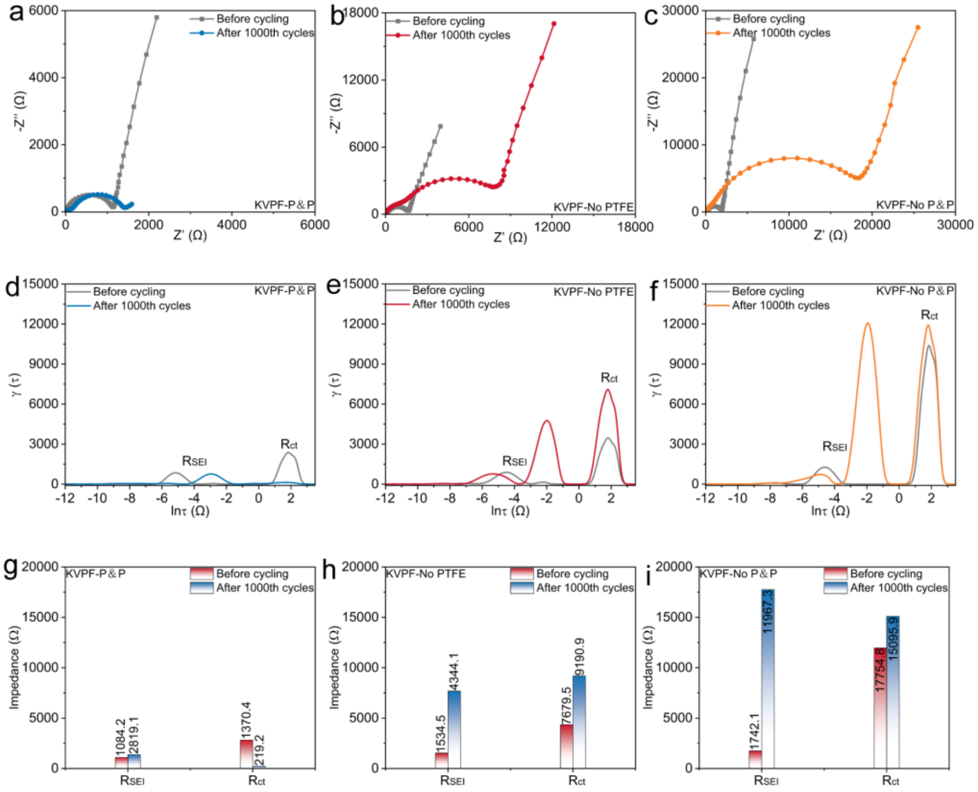

Fig. S34. *Ex-situ* EIS Nyquist plots and corresponding DRTs of (a, d, g) KVPF-P&P, (b, e, h) KVPT-No PTFE and (c, f, i) KVPF-No P&P.

Notes: As shown in **Fig. S34**, both R_{ct} and R_{SEI} of KVPF-P&P remained relatively stable throughout the cycling process, which are obviously lower than those of both KVPT-No PTFE and KVPF-No P&P, indicating that the KVPF-P&P cell maintained excellent kinetics even after 1000 cycles.

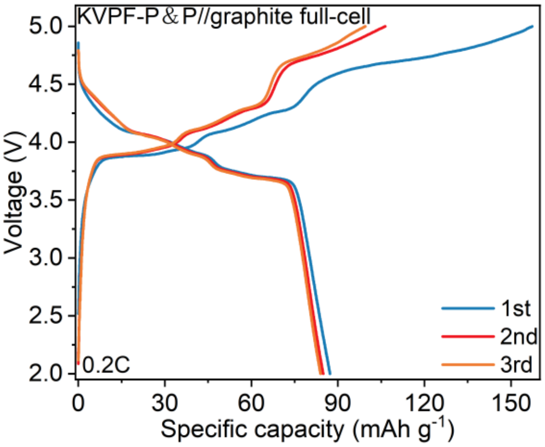

Fig. S35. Galvanostatic charge-discharge curves of KVPF-P&P//graphite full-cell at 0.2C.

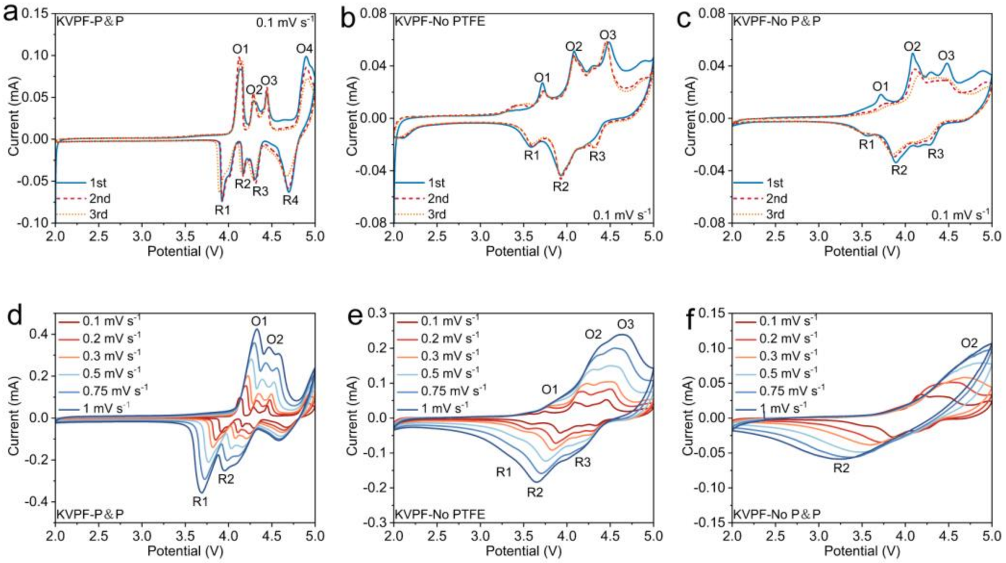

Fig. S36. CV curves of (a) KVPF-P&P, (b) KVPF-PTFE and (c) KVPF-No P&P at 0.1 mV s^{-1}. CV curves of (d) KVPF-P&P, (e) KVPF-PTFE and (f) KVPF-No P&P at selected scan rates.

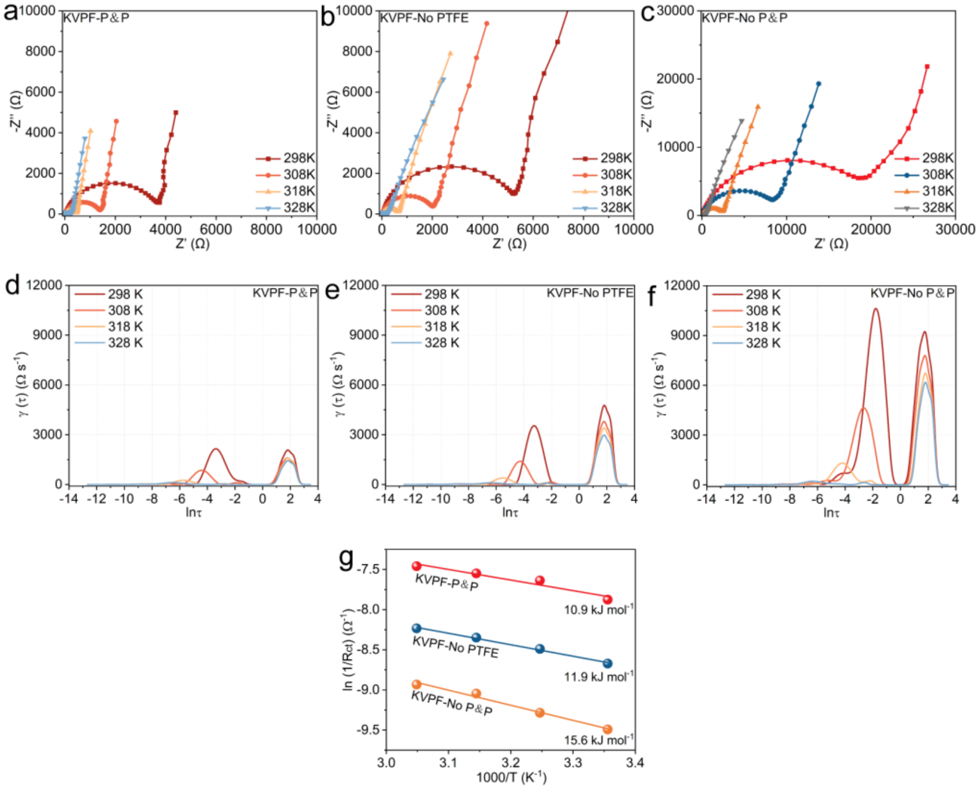

Fig. S37. The temperature dependence EIS Nyquist plots and corresponding calculated DRTs of (a, d) KVPF-P&P, (b, e) KVPF-No PTFE and (c, f) KVPF-No P&P. (g) R_{ct} activation energies.

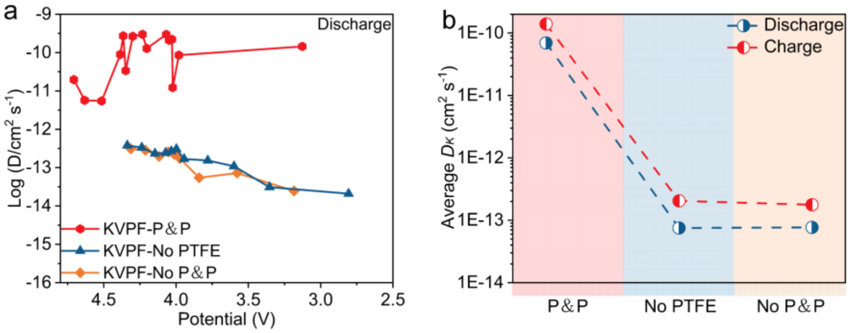

Fig. S38. (a) D_K of KVPF-P&P, KVPF-PTFE and KVPF-No P＆P electrode during the discharge process, (b) The average D_K values of KVPF-P&P, KVPF-PTFE and KVPF-No P＆P electrode.

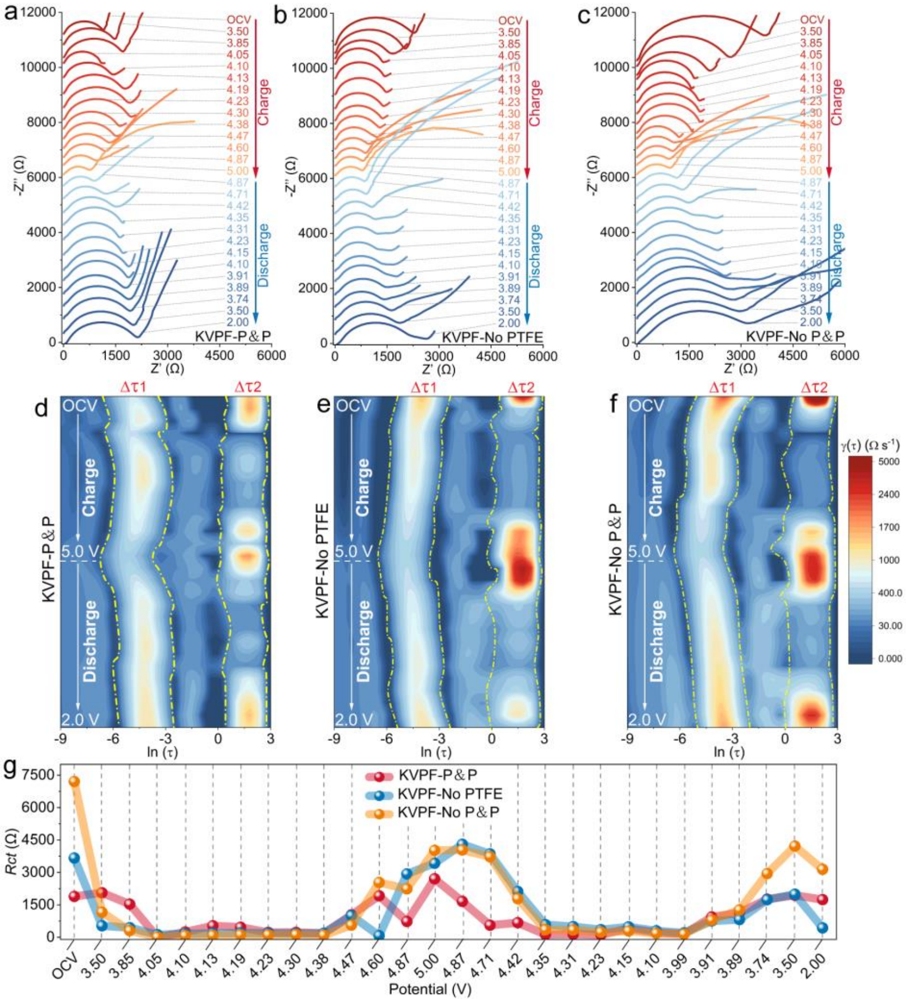

Fig. S39. *In-situ* EIS Nyquist plots and corresponding DRTs of (a, d) KVPF-P&P, (b, e) KVPF-No PTFE and (c, f) KVPF-No P&P electrode. (g) Corresponding calculated impedances at different potentials during the GCD processes.

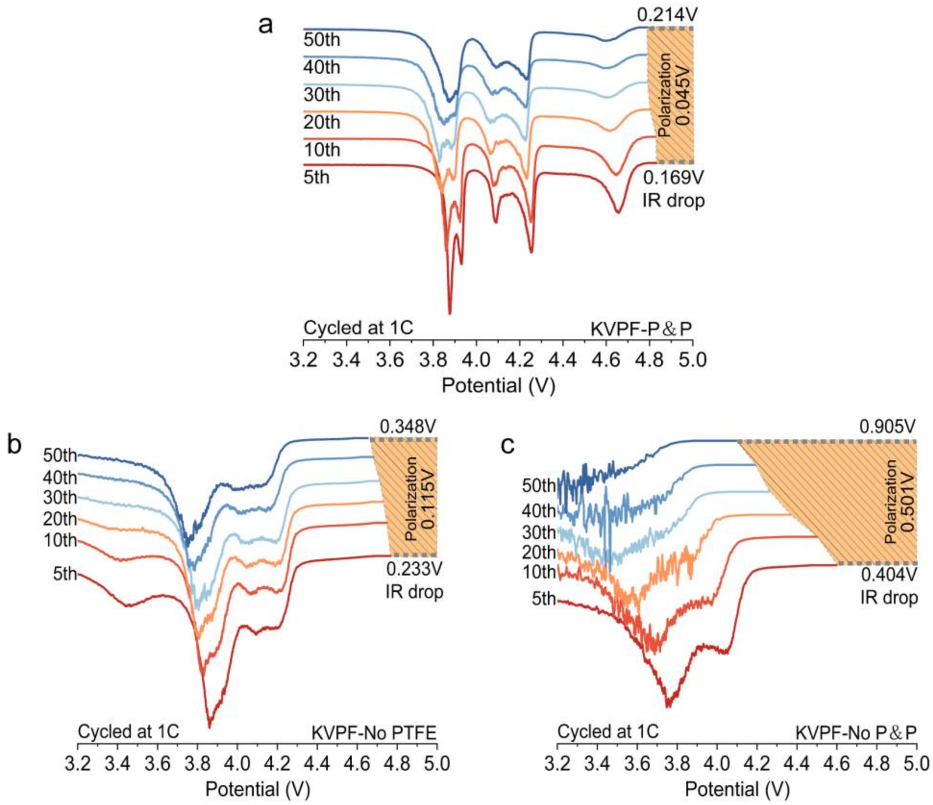

Fig. S40. The dQ/dVcurves for (a) KVPF-P&P, (b) KVPF-No PTFE and (c) KVPF-No P&P electrode.

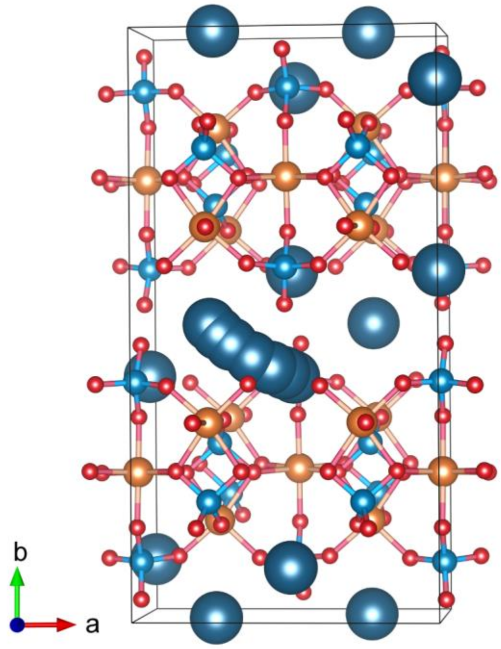

Fig. S41. Schematic illustration of K^+ migration path in layered $K_3V_3(PO_4)_4$.

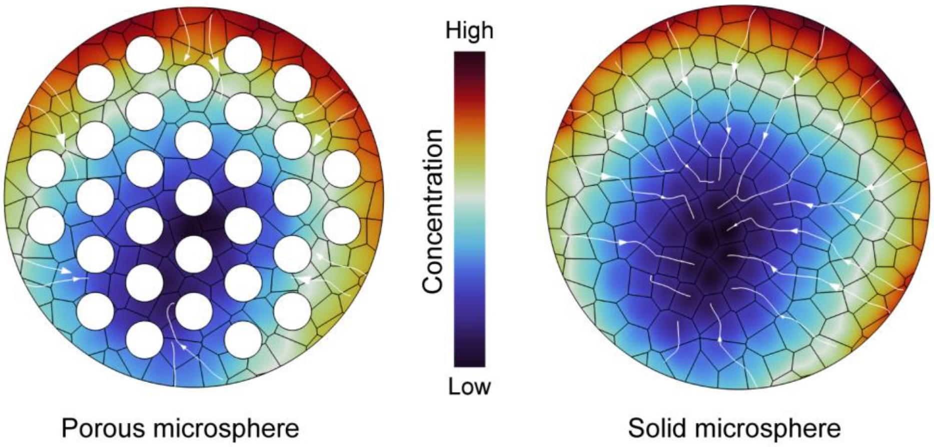

Fig. S42. The simulated results of K^+ concentration distribution in the porous microsphere and solid microsphere.

Table S1. Comparison of electrochemical performance reported for $KVPO_4F$.

Material	Specific capacity (mAh g^{-1})	Rate capacity (mAh g^{-1})	Cycling stability	Working voltage (V)	Electrolyte	Ref.
KVPF-P&P	94.2 (26.2 mA g^{-1})	53.2 (1.31 A g^{-1})	55.4% (1000th, 0.655 A g^{-1})	4.02	0.8M KPF_6 in EC/DEC+1.5wt%TMSPi	This work
$KVPO_4F$	70 (6.65 mA g^{-1})	67.0 (0.655 A g^{-1})	97% (30th, 0.0066 A g^{-1})	4.0	0.7M KPF_6 in EC/DEC	5
$KVPO_4F_{0.5}O_{0.5}@C_2H_2$	84 (26.2 mA g^{-1})	48.1 (1.31 A g^{-1})	88% (20th, 0.0262 A g^{-1})	4.2	0.8M KPF_6 in EC/DEC	6
$KVPO_4F_{0.5}O_{0.5}$	84 (26.2 mA g^{-1})	Failed (1.31 A g^{-1})	Not provided	Not provided		
KVPF-Al	73.3 (26.2 mA g^{-1})	33.6 (1.31 A g^{-1})	41.5% (1000th, 0.655 A g^{-1})	4.01	0.8M KPF_6 in EC/DEC+1.5wt%TMSP	7
KVPF-film	92.4 (26.2 mA g^{-1})	49.0 (1.31 A g^{-1})	74.9% (1000th, 0.655 A g^{-1})	4.02		
KVPF-PVDF	95.8 (26.2 mA g^{-1})	48.3 (1.31 A g^{-1})	64.5% (1000th, 0.655 A g^{-1})	4.02	0.8M KPF_6 in EC/DEC+1.5wt%TMSPi	8
$KVPO_4F$	105 (5 mA g^{-1})	44.0 (0.3 A g^{-1})	78.3% (10th, 0.02 A g^{-1})	4.0	0.7M KPF_6 in EC/DEC	9
$KVPO_{4.36}F_{0.64}$	85 (5 mA g^{-1})	54.0 (0.3 A g^{-1})	91.7% (10th, 0.02 A g^{-1})	Not provided		
KVPF@C-bulk	94.2 (20 mA g^{-1})	21.2 (0.655 A g^{-1})	88.1% (200th, 0.0655 A g^{-1})	4.12	1M KPF_6 in PC/EC	1
KVPF@C-PMS	101.5 (20 mA g^{-1})	70.1 (0.655 A g^{-1})	85.1% (200th, 0.0655 A g^{-1})	4.02		
KVPF	98.6 (20 mA g^{-1})	Failed (0.5 A g^{-1})	71.0% (55th, 0.05 A g^{-1})	4.03	0.8M KPF_6 in EC/DEC	10
KVPF@3DC	103 (20 mA g^{-1})	60.2 (0.5 A g^{-1})	85.4% (550th, 0.5 A g^{-1})	4.03		
KVPF@C-bare	95 (20 mA g^{-1})	88.8 (0.05 A g^{-1})	84.9% (50th, 0.05 A g^{-1})	4.03	0.8M KPF_6 in EC/DEC	11
KVPF@C-MC2	100 (20 mA g^{-1})	84.4 (0.05 A g^{-1})	100% (50th, 0.05 A g^{-1})	4.03		
KVPF@C	82.9 (25 mA g^{-1})	46.3 (1 A g^{-1})	Not provided	Not provided	1M KPF_6 in PC/EC	12
KVPF@K	101.6 (25 mA g^{-1})	47.9 (1 A g^{-1})	53.2% (100th, 0.2 A g^{-1})	3.96		
KVPF@KCO-K	142 (25 mA g^{-1})	70.6 (1 A g^{-1})	74.1% (100th, 0.2 A g^{-1})	3.98		
KVPF-ball	105 (20 mA g^{-1})	59.2 (5 A g^{-1})*	65% (100th, 0.02 A g^{-1})	4.10	0.5M KPF_6 in PC/FEC	13
KVPF-platelet	94 (20 mA g^{-1})	30.0 (5 A g^{-1})*	53% (100th, 0.02 A g^{-1})	4.01		
KVPF-flower	103 (20 mA g^{-1})	87.6 (5 A g^{-1})*	80% (900th, 1 A g^{-1})	3.97		
KVPF-CTR	74.6 (26.2 mA g^{-1})	46.2 (2.62 A g^{-1})**	80.2% (100th, 0.0655 A g^{-1})	4.00	1M KPF_6 in PC/EC+5vol%FEC	14
KVPF@CMK-3	103.2 (26.2 mA g^{-1})	90.1 (2.62 A g^{-1})**	88.2% (500th, 1.31 A g^{-1})	3.93		
KVPF-CTR	94.2 (20 mA g^{-1})	Failed (10 A g^{-1})***	73.5% (100th, 0.05 A g^{-1})	3.96	1M KPF_6 in PC/EC+5wt%FEC	15
KVPF-P3/C	106.5 (20 mA g^{-1})	73.8 (10 A g^{-1})***	82.5% (1000th, 1 A g^{-1})	3.97		
KVPF@rGO	103.2 (20 mA g^{-1})	88.1 (5 A g^{-1}))****	76.9% (500th, 1.31 A g^{-1})	3.94	1M KPF_6 in PC/EC+5vol%FEC	16
KVPF	85.0 (20 mA g^{-1})	26.8 (5 A g^{-1}))****	23.3% (500th, 1.31 A g^{-1})	3.95		

Notes: The test methods for rate performance and cycle stability of some papers are slow charge and fast discharge mode (marked with "*") rather than standard constant current charge/discharge mode, no doubt this will results in significantly improved electrochemical performance.

*The condition of rate performance is 50 mA g^{-1} for charge and 5 A g^{-1} for discharge (slow charging and fast discharging mode). The cycling stability was obtained at a fixed current density of 0.02A g^{-1}.

**The condition of rate performance for KVPF@CMK-3 is 65.5 mA g^{-1} for charge and 1.31 A g^{-1} for discharge (slow charging and fast discharging mode).

***The condition of rate performance for KVPF@P3/C is 65.5 mA g^{-1} for charge and 1 A g^{-1} for discharge (slow charging and fast discharging mode).

****The condition of rate performance for KVPF@RGO is 65.5 mA g^{-1} for charge and 1.31 A g^{-1} for discharge (slow charging and fast discharging mode).

REFERENCES

1. Xie C, Liu XW, Han J *et al.* Pomegranate-like $KVPO_4F$@C microspheres as high-volumetric-energy-density cathode for potassium-ion batteries. *Small* 2022; **18**: 2204348.
2. Yan J, Huang Y, Zhang Y *et al.* Facile synthesis of bimetallic fluoride heterojunctions on defect-enriched porous carbon nanofibers for efficient ORR catalysts. *Nano Lett* 2021; **21**: 2618–24.
3. Zhu Y, Gan L, Shi J *et al.* Co-CoF_2 heterojunctions encapsulated in N, F co-doped porous carbon as bifunctional oxygen electrocatalysts for Zn-air batteries. *Chem Eng J* 2022; **433**: 133541.
4. Hayat MD, Li T, Cao P. Incorporation of PVP into PEG/PMMA based binder system to minimize void nucleation. *Mater Des* 2015; **87**: 932–8.
5. Chihara K, Katogi A, Kubota K *et al.* $KVPO_4F$ and $KVOPO_4$ toward 4 volt-class potassium-ion batteries. *Chem Commun* 2017; **53**: 5208–11.
6. Larbi L, Wernert R, Fioux P *et al.* Enhanced performance of $KVPO_4F_{0.5}O_{0.5}$ in potassium batteries by carbon coating interfaces. *ACS Appl Mater Interfaces* 2023; **15**: 18992–9001.
7. Fu Q, Peng C, Zhou W *et al.* Regulating cathode surface hydroxyl chemistry enables superior potassium storage. *Proc Natl Acad Sci USA* 2023; **120**: e2301622120.
8. Fu Q, Zhou W, Gao P *et al.* Carbene-catalyzed synthesis of a fluorophosphate cathode. *Energy Environ Sci* 2024; **17**: 5147–61.
9. Kim H, Seo DH, Bianchini M *et al.* A new strategy for high-voltage cathodes for K-ion batteries: stoichiometric $KVPO_4F$. *Adv Energy Mater* 2018; **8**: 1801591.
10. Liu ZM, Wang J, Lu BA. Plum pudding model inspired $KVPO_4F$@3DC as high-voltage and hyperstable cathode for potassium ion batteries. *Sci Bull* 2020; **65**: 1242–51.
11. He XD, Zhang LM, Jiang CH *et al.* Elevating cyclability of an advanced $KVPO_4F$ cathode via multi-component coating strategy for high-performance potassium-ion batteries. *Chem Eng J* 2022; **433**: 134634.
12. Heng YL, Gu ZY, Guo JZ *et al.* Low-strain and high-energy $KVPO_4F$ cathode with multifunctional stabilizer for advanced potassium-ion batteries. *Energy Environ Mater* 2024; **7**: e12721.
13. Liao JY, Hu Q, He XD *et al.* A long lifespan potassium-ion full battery based on $KVPO_4F$ cathode and VPO_4 anode. *J Power Sources* 2020; **451**: 227739.
14. Xu J, Duan L, Liao J *et al.* $KVPO_4F$/carbon nanocomposite with highly accessible active sites and robust chemical bonds for advanced potassium-ion batteries. *Green Energy Environ* 2023; **8**: 1469–78.
15. Liao J, Zhang X, Zhang Q *et al.* Synthesis of $KVPO_4F$/carbon porous single crystalline nanoplates for high-rate potassium-ion batteries. *Nano Lett* 2022; **22**: 4933–40.
16. Xu JZ, Liao JY, Xu YF *et al.* Facile synthesis of $KVPO_4F$/reduced graphene oxide hybrid as a high-performance cathode material for potassium-ion batteries. *J Energy Chem* 2022; **68**: 284–92.
